# MHO: A Modified Hippopotamus Optimization Algorithm for Global Optimization and Engineering Design Problems

**DOI:** 10.3390/biomimetics10020090

**Published:** 2025-02-05

**Authors:** Tao Han, Haiyan Wang, Tingting Li, Quanzeng Liu, Yourui Huang

**Affiliations:** School of Electrical & Information Engineering, Anhui University of Science and Technology, Huainan 232001, China; than@aust.edu.cn (T.H.); 2023200859@aust.edu.cn (H.W.); lqz990709@163.com (Q.L.); hyr628@163.com (Y.H.)

**Keywords:** metaheuristic algorithms, hippopotamus optimization, global optimization, engineering design problems

## Abstract

The hippopotamus optimization algorithm (HO) is a novel metaheuristic algorithm that solves optimization problems by simulating the behavior of hippopotamuses. However, the traditional HO algorithm may encounter performance degradation and fall into local optima when dealing with complex global optimization and engineering design problems. In order to solve these problems, this paper proposes a modified hippopotamus optimization algorithm (MHO) to enhance the convergence speed and solution accuracy of the HO algorithm by introducing a sine chaotic map to initialize the population, changing the convergence factor in the growth mechanism, and incorporating the small-hole imaging reverse learning strategy. The MHO algorithm is tested on 23 benchmark functions and successfully solves three engineering design problems. According to the experimental data, the MHO algorithm obtains optimal performance on 13 of these functions and three design problems, exits the local optimum faster, and has better ordering and stability than the other nine metaheuristics. This study proposes the MHO algorithm, which offers fresh insights into practical engineering problems and parameter optimization.

## 1. Introduction

Finding the maximum value of a given objective function under specified constraints is the goal of optimization problems, which are found in a variety of disciplines, including computer science, mathematics, engineering, and economics. All optimization problems consist of three components: the objective function, constraints, and decision variables [1].

Traditional optimization algorithms, such as linear programming, quadratic programming, dynamic programming, etc., provide a solid mathematical foundation and efficient solutions for solving deterministic, convex, and well-structured optimization problems, but they usually require the problem to have a specific mathematical structure, and they are prone to fall into locally optimal solutions, especially in the case of multi-peak problems, where it is difficult to find the globally optimal solution, and the results of the solution strongly depend on the initial values [2]. The rise of metaheuristic algorithms compensates for the limitations of conventional optimization algorithms, as they are very flexible and adaptable and offer new tools and techniques for resolving challenging optimization issues in the real world. These algorithms are independent of the problem’s form and do not require knowledge of the objective function’s derivatives [3].

Metaheuristics are high-level algorithms that model social behaviors or natural phenomena to discover an approximate optimal solution to complex optimization problems. There are a wide variety of metaheuristic algorithms, which can be categorized into three groups based on their inspiration and working principles: evolution-based algorithms, group intelligence-based algorithms, and algorithms based on physical principles [4]. Evolution-based algorithms are mainly used to realize the overall progress of the population and finally complete the optimal solution by simulating the evolutionary law of superiority and inferiority in nature (Darwin’s law) [5]. Among the most prominent examples of these are genetic algorithms (GA) [6] and differential evolution (DE) [7]. Genetic algorithms simulate the process of biological evolution and optimize the solution through selection, crossover and mutation operations, with strong global search abilities which are suitable for discrete optimization problems. Differential evolution algorithms generate new solutions through the different operations of individuals in a population, which is excellent in dealing with nonlinear and multimodal optimization problems. By simulating a group’s intelligence, group intelligence-based algorithms [8,9] aim to produce a globally optimal solution. Each group in this algorithm is a biological population, and the most representative examples are the particle swarm optimization algorithm [10] and the ant colony algorithm [11], which use the cooperative behavior of a population to accomplish tasks that individuals are unable to complete. The PSO simulates the social behavior of bird or fish flocks and achieves global optimization through collaboration among individuals, which is simple, efficient, and suitable for continuous optimization problems. The ACO simulates the foraging behavior of ants and optimizes the paths through a pheromone mechanism, which is excellent in path optimization problems. There are also many other popular algorithms, such as the artificial bee colony algorithm [12], which simulates the foraging behavior of bees to optimize solutions through information sharing and collaboration, the bat optimization algorithm [13], which simulates the echolocation behavior of bats to optimize solutions through frequency and amplitude adjustment, and the gray wolf optimization algorithm [14], which simulates the collaboration and competition between leaders and followers in gray wolf packs. All of these algorithms have strong global search capabilities. The firefly algorithm (FA), which simulates the behavior of fireflies glowing to attract mates, optimizes solutions through light intensity and movement rules for multi-peak optimization problems. The fundamental concept of physical principle-based algorithms, of which simulated annealing (SA) [15] is the best example, is to use natural processes or physics principles as the basis for search techniques used to solve complex optimization problems. It mimics the annealing process of solids and performs well in combinatorial optimization problems by controlling the “temperature” parameter to balance global exploration and local exploitation in the search process. In addition to the above algorithms, others include the gravitational search algorithm (GSA) [16] and the water cycle algorithm (WCA) [17]. The GSA optimizes the solution by simulating gravitational interactions between celestial bodies and using mutual attraction between masses, demonstrating a strong global search capability. The WCA, on the other hand, simulates water cycle processes in nature and uses the convergence and dispersion mechanism of water flow to optimize the solution, which also has excellent global search performance. In addition, there are special types of hybrid optimization algorithms, which combine the features of two or more metaheuristics to enhance the performance of the algorithms by incorporating different search mechanisms. For example, the hybrid particle swarm optimization algorithm with differential evolution (DEPSO [18]) combines the population intelligence of the particle swarm optimization algorithm and the variability capability of differential evolution, which enables DEPSO to efficiently balance global and local searches and to improve the efficiency and effectiveness of the optimization process, especially for global optimization problems in continuous space. Based on a three-phase model that includes hippopotamus positioning in rivers and ponds, defense strategies against predators, and escape strategies, the HO is a new algorithm inspired by hippopotamus population behaviors which was proposed by Amiri [19] et al. in 2024. In the optimization sector, the hippopotamus optimization (HO) algorithm stands out for its excellent performance, which is able to quickly identify and converge to the optimal solution and effectively avoid falling into local minima. The algorithm’s efficient local search strategy and fast optimality-finding speed enable it to excel in solving complex problems. It effectively balances global exploration and local exploitation and is able to quickly find high-quality solutions, making it an effective tool for solving complex optimization problems.

Currently, metaheuristic algorithms have a wide range of application prospects in the field of engineering optimization. Hu [20] et al. used four metaheuristic algorithms, namely, the African vulture optimization algorithm (AVOA), the teaching–learning-based optimization algorithm (TLBO), the sparrow search algorithm (SSA), and the gray wolf optimization algorithm (GWO), to optimize a hybrid model and proposed integrated prediction of steady-state thermal performance prediction data for an energy pile-driven model. Sun [21] et al. responded to most of the industrial design problems and proposed a fuzzy logic particle swarm optimization algorithm based on the associative constraints processing method. A particle swarm optimization algorithm was used as a searcher, and a set of fuzzy logic rules integrating the feasibility of the individual was designed to enhance its searching ability. Wu [22] et al. responded to the ant colony optimization algorithm’s limitations, such as early blind searching, slow convergence, low path smoothness, and other limitations, and proposed an ant colony optimization algorithm based on farthest point optimization and a multi-objective strategy. Palanisamy and Krishnaswamy [23] used hybrid HHO-PSO (hybrid particle swarm optimization) for failure testing of wire ropes for hardness, wear and tear analysis, tensile strength, and fatigue life and adopted a hybrid HHO-based artificial neural network-based HHO (Hybrid ANN-HHO) to predict the performance of the experimental wire ropes. Liu [24] et al. proposed an improved adaptive hierarchical optimization algorithm (HSMAOA) in response to problems such as premature convergence and falling into local optimization when dealing with complex optimization problems in arithmetic optimization algorithms. Cui [25] et al. combined the whale optimization algorithm (WOA) with attention-to-the-technology (ATT) and convolutional neural networks (CNNs) to optimize the hyperparameters of the LSTM model and proposed a new load prediction model to address the over-reliance of most methods on the default hyperparameter settings. Che [26] et al. used a circular chaotic map as well as a nonlinear function for multi-strategy improvement of the whale optimization algorithm (WOA) and used the improved WOA to optimize the key parameters of the LSTM to improve its performance and modeling time. Elsisi [27] used a different learning process based on the improved gray wolf optimizer (IGWO) and fitness–distance balancing (FDB) methodology to balance the original gray wolf optimizer’s exploration and development approach and design a new automated adaptive model predictive control (AMPC) for self-driving cars to solve the rectification problem of self-driving car parameters and the uncertainty of the vision system. Karaman [28] et al. used the artificial bee colony (ABC) optimization algorithm to go in search of the optimal solution for the hyperparameters and activation function of the YOLOv5 algorithm and enhance the accuracy of colonoscopy. Yu and Zhang [29], in order to minimize the wake flow effect, proposed an adaptive moth flame optimization algorithm with enhanced detection exploitation capability (MFOEE) to optimize the turbine layout of wind farms. Dong [30] et al. optimized the genetic algorithm (GA) based on the characteristics of flood avoidance path planning and proposed an improved ant colony genetic optimization hybrid algorithm (ACO-GA) to achieve dynamic planning of evacuation paths for dam-breaking floods. Shanmugapriya [31] et al. proposed an IoT-based HESS energy management strategy for electric vehicles by optimizing the weight parameters of a neural network using the COA technique to improve the SAGAN algorithm in order to improve the battery life of electric vehicles. Beşkirli and Dağ [32] proposed an improved CPA algorithm (I-CPA) based on the instructional factor strategy and applied it to the problem of solar photovoltaic (PV) module parameter identification in order to improve the accuracy and efficiency of PV model parameter estimation. Beşkirli and Dağ [33] proposed a multi-strategy-based tree seed algorithm (MS-TSA) which effectively improves the global search capability and convergence performance of the algorithm by introducing an adaptive weighting mechanism, a chaotic elite learning method, and an experience-based learning strategy. It performs well in both CEC2017 and CEC2020 benchmark tests and achieves significant optimization results in solar PV model parameter estimation. Liu [34] et al. proposed an improved DBO algorithm and applied it to the optimal design of off-grid hybrid renewable energy systems to evaluate the energy cost with life cycle cost as the objective function. However, the above algorithms face the challenges of data size and complexity in practical applications and still suffer from the problem of easily falling into local optima, low efficiency, and insufficient robustness, which limit the performance and applicability of the algorithms.

When solving real-world problems, the HO algorithm excels due to its adaptability and robustness and is able to maintain stable performance in a wide range of optimization problems, making it an ideal choice for fast and efficient optimization problems. Maurya [35] et al. used the hippopotamus optimization algorithm (HO) to optimize distributed generation planning and network reconfiguration in the consideration of different loading models in order to improve the performance of a power grid. Chen [36] et al. addressed the limitations of the VMD algorithm and improved it by using the excellent optimization capability of the HO algorithm to achieve preliminary denoising, and in doing so, proposed a single-sign-on modal identification method based on hippopotamus optimization-variational modal decomposition (HO-VMD) and singular value decomposition-regularized total least squares-Prony (SVD-RTLS-Prony) algorithms. Ribeiro and Muñoz [37] used particle swarm optimization, hippopotamus optimization, and differential evolution algorithms to tune a controller with the aim of minimizing the root mean square (RMS) current of the batteries in an integrated vehicle simulation, thus mitigating battery stress events and prolonging its lifetime. Wang [38] et al. used an improved hippopotamus optimization algorithm (IHO) to improve solar photovoltaic (PV) output prediction accuracy. The IHO algorithm addresses the limitations of traditional algorithms in terms of search efficiency, convergence speed, and global searching. Mashru [39] et al. proposed the multi-objective hippopotamus optimizer (MOHO), which is a unique approach that excels in solving complex structural optimization problems. Abdelaziz [40] et al. used the hippopotamus optimization algorithm (HO) to optimize two key metrics and proposed a new optimization framework to cope with the problem of the volatility of renewable energy generation and unpredictable electric vehicle charging demand to enhance the performance of the grid. Baihan [41] et al. proposed an optimizer-optimized CNN-LSTM approach that hybridizes the hippopotamus optimization algorithm (HOA) and the pathfinder algorithm (PFA) with the aim of improving the accuracy of sign language recognition. Amiri [42] et al. designed and trained two new neuro-fuzzy networks using the hippopotamus optimization algorithm with the aim of creating an anti-noise network with high accuracy and low parameter counts for detecting and isolating faults in gas turbines in power plants. In addition to the above applications, there are many global optimization and engineering design problems. However, the theory of “no-free-lunch” (NFL) states that no optimization algorithm can solve all problems [43], and each existing optimization algorithm can only achieve the expected results on certain types of problems, so improvement of the HO algorithm is still necessary. Although the HO algorithm has many advantages, its performance level decreases when dealing with complex global optimization and engineering design problems, and it cannot avoid falling into local optima. It is still necessary to adjust the algorithm parameters and strategies according to specific problems in practical applications in order to fully utilize its potential. Therefore, we propose the MHO algorithm to enhance the ability of HO to solve these problems. The main contributions of this paper are as follows:

- Use the method of the sine chaotic map to replace the original population initialization method in order to prevent the HO algorithm from settling into local optimal solutions and to produce high-quality starting solutions.
- Introduce a new convergence factor to alter the growth mechanism of hippopotamus populations during the exploration phase improves the global search capability of HO.
- Incorporate a small-hole imaging reverse learning strategy into the hippopotamus escaping predator stage to avoid interference between dimensions, expand the search range of the algorithm to avoid falling into a local optimum, and thus improve the performance of the algorithm.
- The MHO model is tested on 23 benchmark functions, the optimization ability of the model is tested by comparing it with other algorithms, and three engineering design problems are successfully solved.

The structure of this paper is as follows: Section 2 presents the hippopotamus algorithm and three methods for enhancing the hippopotamus optimization algorithm; Section 3 presents experiments and analysis, including evaluating the experimental results and comparing the MHO algorithm with other algorithms; Section 4 applies MHO to three engineering design problems; and Section 5 provides a summary of the entire work.

## 2. Improved Algorithm

### 2.1. Sine Chaotic Map

A sine chaotic map [44] is a kind of chaotic system that generates chaotic sequences by nonlinear transformation of a sinusoidal function, which becomes a typical representative of a chaotic map due to the advantages of simple structure and high efficiency, and its mathematical expression is

(1) $$x_{k+1}=\alpha sin\left(x_{k}\right)$$

where k is a non-negative integer; $x_{k}\in \left[0,1\right]$ denotes the value of the current iteration step; and $\alpha \in \left[0,1\right]$ is the chaos coefficient control parameter.

**Figure 1:**
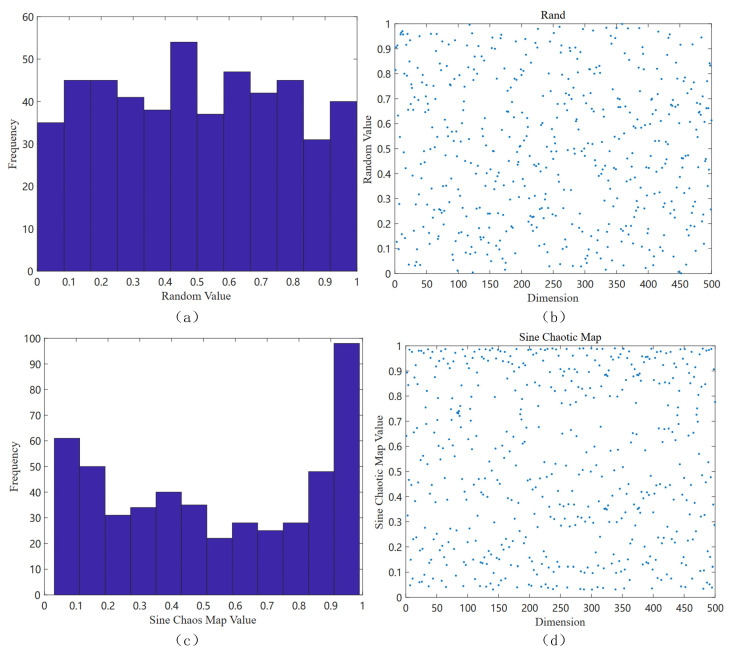
Comparison of the distribution of algorithmic initialization: (**a**) histogram of frequency distribution of conventional random initialization; (**b**) scatter plot of the distribution of conventional random initialization in two-dimensional space; (**c**) histogram of frequency distribution of sinusoidal chaotic map initialization; and (**d**) scatter plot of the distribution of sinusoidal chaotic map initialization in two-dimensional space.

The sine map starts chaotic behavior when the parameter α is close to 0.87, and superior chaotic properties can be observed when α is close to 1. Therefore, the introduction of the sine chaotic map into the random initialization of the initial value of the hippopotamus optimization (HO) algorithm can make the hippopotamus population uniformly distributed throughout the search space, which improves the diversity of the initial population, enhances the global search capability of the HO algorithm, and effectively avoids falling into the local optimal solution. Figure 1 shows the population distribution initialized by the algorithm:

In the HO algorithm, a hippopotamus is a candidate solution to the optimization problem, which means that each hippopotamus’ position in the search space is updated to represent the values of the decision variables. Thus, each hippopotamus is represented as a vector and the population of hippopotamuses is mathematically characterized by a matrix. Similar to traditional optimization algorithms, the initialization phase of HO involves the generation of a random initial solution, and the vector of decision variables is generated as follows:

(2) $$X_{i}:x_{i,j}=lb_{j}+r\times \left(ub_{j}-lb_{j}\right),\;i=1,2,\ldots ,N;\;j=1,2,\ldots ,m$$

where $X_{i}$ denotes the location of the ith candidate solution, r is a random number in the range of 0~1, and lb and ub represent the lower and upper limits of the jth decision variable, respectively. Let N denote the population size of hippopotamus in the herd, while m denotes the number of decision variables in the problem and the population matrix is composed by Equation (3).

(3) $$x={\left[\begin{array}{c} x_{1}\\ \vdots \\ x_{i}\\ \vdots \\ x_{N} \end{array}\right]}_{N\times m}={\left[\begin{array}{ccccc} x_{1,1} & \cdots & x_{1,j} & \cdots & x_{1,m}\\ \vdots & \ddots & \vdots & ⋰ & \vdots \\ x_{i,1} & \cdots & x_{i,j} & \cdots & x_{i,m}\\ \vdots & ⋰ & \vdots & \ddots & \vdots \\ x_{N,1} & \cdots & x_{N,j} & \cdots & x_{N,m} \end{array}\right]}_{N\times m}$$

Consequently, the improved expression for the initialization phase is

(4) $$X_{i}:x_{i,j}=lb_{j}+Sine\_chaos\left(ub_{j}-lb_{j}\right)$$

and furthermore,

(5) $$Sine\_chaos=\alpha sin\left(k\pi x\right)$$

where k is a parameter that controls the chaotic behavior and x is an initial value.

### 2.2. Change Growth Mechanism

The growth mechanism is a key component of the hippopotamus optimization algorithm that determines how the search strategy is updated to find better solutions based on current information.

In the original growth mechanism, the exploration phase of the HO algorithm models the activity of the hippopotamus itself in the entire herd. The authors subdivided the whole population into four segments, i.e., adult female hippopotamus, young hippopotamus, adult male hippopotamus, and the dominant male hippopotamus (the leader of the herd). The dominant hippopotamus was determined iteratively based on the value of the objective function (minimizing the minimum value of the problem and maximizing the maximum value of the problem).

In a typical hippopotamus herd, several females are positioned around the males, and the herd leader defends the herd and territory from possible attacks. When hippopotamus calves reach adulthood, the dominant male ejects them from the herd. Subsequently, these expelled males are asked to attract females or compete for dominance with other established male members. The location of the herd’s male hippopotamus in a lake or pond is represented mathematically by Equation (6).

(6) $$X_{i}^{\mathrm{Mhipoo}}:x_{i}^{\mathrm{Mhippo}}=x_{ij}+y_{1}\left(D_{\mathrm{hippo}}-I_{1}x_{ij}\right)$$

In Equation (6), $X_{i}^{\mathrm{Mhipoo}}$ denotes the position of the male hippopotamus and $D_{\mathrm{hippo}}$ indicates the location of the dominant hippopotamus. As shown in Equation (7), ${\vec{r}}_{1,\ldots ,4}$ is a random vector between 0 and 1, $r_{5}$ is a random number between 0 and 1, $I_{1}$ and $I_{2}$ are integers between 1 and 2. $MG_{i}$ is the average of a number of randomly selected hippopotamuses, which includes the currently considered hippopotamus with equal probability, $y_{1}$ is a random number between 0 and 1, and $e_{1}$ and $e_{2}$ are random integers that can be either 1 or 0.


(7) $$h=\left\{\begin{array}{c} \begin{array}{c} I_{2}\times {\vec{r}}_{1}+\left(\sim e_{1}\right)\\ 2\times {\vec{r}}_{2}-1 \end{array}\\ {\vec{r}}_{3}\\ \begin{array}{c} I_{1}\times {\vec{r}}_{4}+\left(\sim e_{2}\right)\\ r_{5} \end{array} \end{array}\right.$$

(8) $$T=\exp\left(-\frac{t}{Max\_iterations}\right)$$

(9) $$X_{i}^{FB\mathrm{hippo}}:x_{i}^{FB\mathrm{hippo}}=\left\{\begin{array}{cc} x_{ij}+h_{1}\cdot \left(D_{\mathrm{hippo}}-I_{2}MG_{i}\right) & ,T>0.6\\ \Xi & ,else \end{array}\right.$$

(10) $$\begin{array}{l} \Xi =\left\{\begin{array}{cc} x_{ij}+h_{2}\cdot \left(MG_{i}-D_{\mathrm{hippo}}\right) & ,r_{6}>0.5\\ lb_{j}+r_{7}\left(ub_{j}-lb_{j}\right) & ,else \end{array}\right.\\ for\;i=1,2,\ldots ,\left[\frac{N}{2}\right]\;\mathrm{and}\;j=1,2,\ldots ,m \end{array}$$

Equations (9) and (10) describe the position of the female or immature hippopotamus in the herd ($X_{i}^{FBhippo}$). The majority of immature hippos are with their mothers, but due to curiosity, sometimes immature hippos are separated from the herd or stay away from their mothers.

If the convergence factor T is greater than 0.6, this means that the immature hippo has distanced itself from its mother (Equation (9)). If $r_{6}$ is greater than 0.5, this means that the immature hippopotamus has distanced itself from its mother but is still in or near the herd; otherwise, it has left the herd. Equations (9) and (10) are based on modeling this behavior for immature and female hippos. Randomly chosen numbers or vectors, denoted as $I_{1}$ and $I_{2}$, are extracted from the set of five scenarios outlined in equation h. In Equation (10), $r_{7}$ is a random number between 0 and 1. Equations (11) and (12) describe the position update of female or immature hippos. The objective function value is denoted by $F_{i}$:

(11) $$X_{i}=\left\{\begin{array}{cc} X_{i}^{M\mathrm{hippo}}F_{i}^{M\mathrm{hippo}} & <F_{i}\\ X_{i} & \mathrm{else} \end{array}\right.$$

(12) $$X_{i}=\left\{\begin{array}{c} X_{i}^{FB\mathrm{hippo}}F_{i}^{FB\mathrm{hippo}}\\ X_{i} \end{array}\right.$$

Using h-vectors, $I_{1}$ and $I_{2}$ scenarios enhance the algorithm’s global search and improve its exploration capabilities. 

The growth mechanism is improved by introducing a new convergence factor T, which is specifically designed to dynamically adjust the behavioral patterns of immature hippos, and the following equation is an improved formulation of T:

(13) $$T=1-{\left(\frac{t}{Max\_iterations}\right)}^{6}$$

(14) $$X_{i}^{FB\mathrm{hippo}}:x_{ij}^{FB\mathrm{hippo}}=\left\{\begin{array}{cc} x_{ij}+h_{1}\cdot \left(D_{\mathrm{hippo}}-I_{2}MG_{i}\right) & ,T>0.95\\ \Xi & ,else \end{array}\right.$$

(15) $$\begin{array}{l} \Xi =\left\{\begin{array}{cc} x_{ij}+h_{2}\cdot \left(MG_{i}-D_{\mathrm{hippo}}\right) & ,r_{6}>0.5\\ lb_{j}+r_{7}\cdot \left(ub_{j}-lb_{j}\right) & ,else \end{array}\right.\\ for\;i=1,2,\ldots ,\left[\frac{N}{2}\right]\;\mathrm{and}\;j=1,2,\ldots ,m \end{array}$$

where t is the current iteration number and Max_iterations is the maximum iteration numbers.

**Figure 2:**
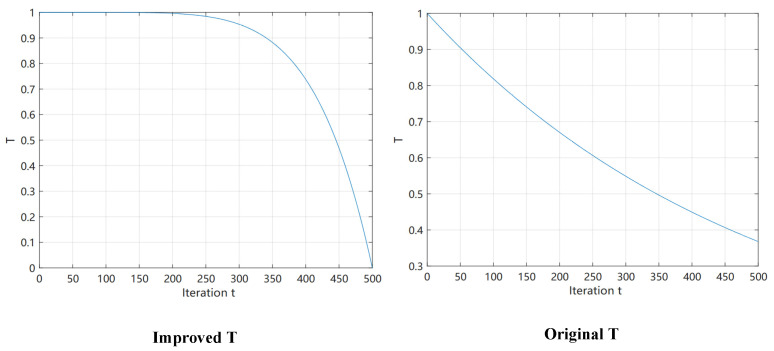
Plots of convergence factor T before and after improvement.

Plots of the functions of Equations (8) and (13) before and after the improvement are shown in Figure 2. The simulated immature hippopotamus individuals will show a higher propensity to explore within the hippopotamus population or within the surrounding area when T>0.95 (Equation (14)). This behavior promotes the algorithm to refine its search in a local region close to the current optimal solution, thus enhancing the algorithm’s search accuracy and efficiency in that region. The immature hippo attempts to move away from the present optimal solution when T≤0.95 and $r_{6}>0.5$. This is a method intended to prolong the search in order to lower the possibility that the algorithm would fall into a local optimum and to enable a more thorough investigation of the global solution space (Equation (15)). The algorithm is able to identify and escape potential local optimality traps more efficiently this way, thus increasing the probability of finding a globally optimal solution. When $r_{6}\leq 0.5$, immature hippos perform random exploration, allowing the algorithm to maintain diversity and avoid premature convergence. This improvement enhances the HO algorithm’s search capability and adaptability by better simulating the natural behavior of hippos.

### 2.3. Small-Hole Imaging Reverse Learning Strategy

Many academics have proposed the reverse learning strategy to address the issue that most intelligent optimization algorithms are prone to local extremes [45]. The core idea behind this strategy is to create a corresponding reverse solution for the current solution during population optimization, compare the objective function values of these two solutions, and choose the better solution to move on to the next iteration. Based on this approach, this study presents small-hole imaging reverse learning [46] technique to enhance population variety, which enhances the algorithm’s global search capability and more accurately approximates the global optimal solution. 

**Figure 3:**
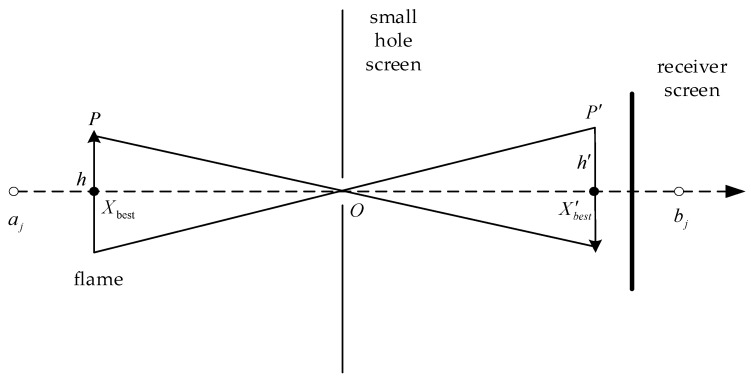
Schematic diagram of small-hole imaging reverse learning.

The principle of small-hole imaging is shown in Figure 3, which is a combined method combining pinhole imaging with dimension-by-dimension inverse learning derived from LensOBL [47]. The aim is to find an inverse solution for each dimension of the feasible solution, thus reducing the risk of the algorithm falling into a local optimum.

Assume that in a certain space, there is a flame p with height h whose projection on the *X*-axis is $X_{\mathrm{best}}^{j}$ (the jth dimensional optimal solution), the upper and lower bounds of the coordinate axes are $a_{j}$ and $b_{j}$ (the upper and lower bounds of the jth dimensional solution), and a screen with a small hole is placed on the base O. The flame passing through the small hole can receive an inverted image $p^{\prime }$ with height $h^{\prime }$ on the receiving screen. The flame passing through the small hole can receive an inverted image $p^{\prime }$ of height $h^{\prime }$ on the receiving screen, and then a reversed point ${X^{\prime }}_{\mathrm{best}}$ (the reversed solution of the jth dimensional solution) is obtained on the *X*-axis through small-hole imaging. Therefore, from the principle of small-hole imaging, Equation (16) can be derived.


(16) $$\frac{\frac{\left(a_{j}-b_{j}\right)}{2}-X_{\mathrm{best}}}{{X^{\prime }}_{\mathrm{best}}-\frac{\left(a_{j}-b_{j}\right)}{2}}=\frac{h}{h^{\prime }}$$

Let $\frac{h}{h^{\prime }}=n$; through the transformation to obtain ${X^{\prime }}_{\mathrm{best}}$, the expression is Equation (17), and Equation (18) is obtained when n=1.

(17) $${X^{\prime }}_{\mathrm{best}}=\frac{\left(a_{j}+b_{j}\right)}{2}+\frac{\left(a_{j}+b_{j}\right)}{2n}-\frac{X_{\mathrm{best}}}{n}$$

(18) $${X^{\prime }}_{\mathrm{best}}=\left(a_{j}+b_{j}\right)-X_{\mathrm{best}}$$

As can be seen from Equation (18), small-hole imaging reverse learning is the correct general reverse learning strategy when n=1, but at this time, small-hole imaging learning is only the current optimal position through general reverse learning to obtain a fixed reverse point; this fixed position is frequently far away from the global optimal position. Therefore, by adjusting the distance between the receiving screen and the small-hole screen to change the adjustment factor n, we can use the algorithm to obtain an optimal solution closer to the position, making it jump out of the local optimal region and closer to the global optimal region.

The development phase of the original hippopotamus algorithm describes a hippopotamus fleeing from a predator. Another behavior of a hippopotamus facing a predator occurs when a hippopotamus is unable to repel a predator with its defensive behaviors, so the hippopotamus tries to get out of the area in order to avoid the predator. This strategy causes the hippo to find a safe location close to its current position. In the third phase, the authors simulate this behavior, which improves the algorithm’s local search capabilities. Random places are created close to the hippo’s present location in order to simulate this behavior.

(19) $$\begin{array}{l} X_{i}^{\mathrm{HippoE}}:x_{ij}^{\mathrm{HippoE}}=x_{ij}+r_{10}\cdot \left(lb_{j}^{\mathrm{local}}+s_{1}\cdot \left(ub_{j}^{\mathrm{local}}-lb_{j}^{\mathrm{local}}\right)\right)\\ \left(i=1,2,\ldots ,N\cdot j=1,2,\ldots ,m\right) \end{array}$$

(20) $$lb_{j}^{\mathrm{local}}=\frac{lb_{j}}{t},\;ub_{j}^{\mathrm{local}}=\frac{ub_{j}}{t},\;t=1,2,\ldots ,\tau .$$

(21) $$s=\left\{\begin{array}{c} 2\times \vec{r_{11}}-1\\ r_{12}\\ r_{13} \end{array}\right.$$

where $X_{i}^{\mathrm{Hippo}\varepsilon }$ is the position of the hippo when it escaped from the predator, and it is searched to find the closest safe position. Out of the three s situations, $s_{1}$ is a randomly selected vector or number (Equation (21)). Better localized search is encouraged by the possibilities that the s equations take into account, and $r_{11}$ represents a random vector between 0 and 1, while $r_{10}$ and $r_{13}$ denote random numbers generated in the range of 0 to 1. In addition, $r_{12}$ is a normally distributed random number. t denotes the current iteration number, while τ denotes the highest iteration number.


(22) $$X_{i}=\left\{\begin{array}{ll} X_{i}^{\mathrm{Hippo}\varepsilon } & ,F_{i}^{\mathrm{Hippo}\varepsilon }<F_{i}\\ X_{i} & ,F_{i}^{\mathrm{Hippo}\varepsilon }\geq F_{i} \end{array}\right.$$

The fact that the fitness value improved at the new position suggested that the hippopotamus had relocated to a safer area close to its original location.

Incorporating the small-hole imaging reverse learning strategy into the HO algorithm can effectively improve the diversity and optimization efficiency of the algorithm. This strategy enhances population diversity and expands the search range through chaotic sequences while mapping the optimal solution dimension by dimension to reduce interdimensional interference and improve global search capability. Additionally, it enhances stability, lowers the possibility of a local optimum, dynamically modifies the search range, and synchronizes the global search with the local exploitation capabilities, all of which help the algorithm to find a better solution with each iteration.

### 2.4. Algorithmic Process

**Figure 4:**
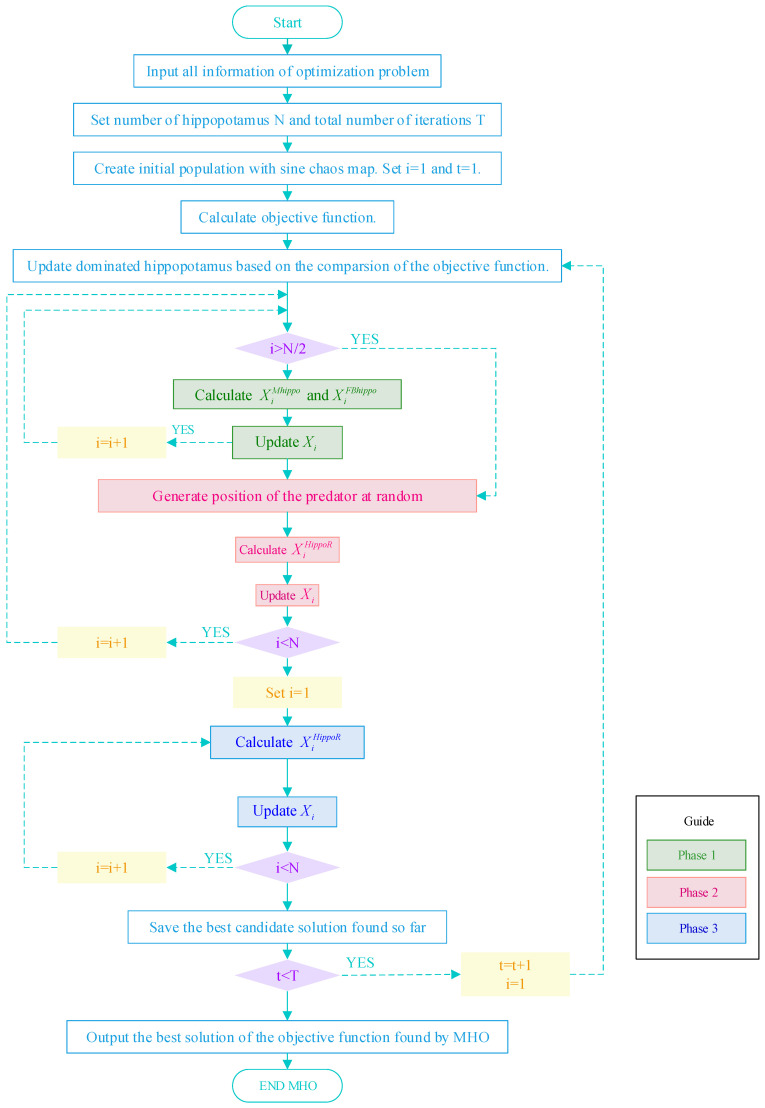
Flowchart of MHO algorithm.

The program details of MHO are shown in the flowchart in Figure 4. Firstly, create an initial population using the sine chaotic map, and set the iteration counter to i=1 and the time counter t=1. Secondly, it is divided into three phases: When i≤N/2, enter the first phase (Phase 1), which is the position update of the hippopotamus in the river or pond (exploration phase). Use Equations (9) and (14) to calculate the positions of male and female hippos, respectively, and update the positions of the hippos using Equations (11) and (12). When i>N/2, it enters the second phase (phase 2), i.e., hippopotamus defense against predators, which is consistent with the original hippopotamus algorithm. The third phase begins when i>N, where the hippopotamus escapes from the predator, and the final position of the hippopotamus is calculated and the hippo’s nearest safe position is updated using Equation (17). Finally, if the time counter is at t<T, increase t and reset the iteration counter i=1 to continue the iteration. The optimal objective function solution discovered by the MHO algorithm is output once the maximum number of iterations T has been reached.

### 2.5. Computational Complexity

Time complexity is a basic index to evaluate the efficiency of algorithms, which is analyzed in this paper by using the method of BIG-O [48]. Assuming that the population size is P, the dimension is D, and the number of iterations is T, the time complexities of the HO algorithm and the MHO algorithm are analyzed as follows:

The standard HO algorithm consists of two phases: a random population initialization phase and a subsequent hippo position update. In the initialization phase, the time complexity of HO can be expressed as $T_{1}=O\left(P\times D\right)$. In the position updating phase, the hippopotamus employs position updating in rivers or ponds for defense and escape from predatory mechanisms. The computational complexity of each iteration is $O\left(P\times D\right)$, and after T iterations, the computational complexity accumulates as $T_{2}=O\left(T\times P\times D\right)$. Therefore, the total time complexity of HO can be expressed as $T_{HO}=T_{1}+T_{2}$ with $O\left(P\right)$ time complexity.

The proposed MHO algorithm consists of three phases: population initialization based on sine chaotic mapping, hippopotamus position updating, and the small-hole imaging reverse learning phase. In the sine chaotic mapping-based population initialization phase, the time complexity of the MHO initialization is denoted as $T_{1}^{\prime }$ and is expressed as $T_{1}^{\prime }=O\left(P\times D\right)$. The hippopotamus position update phase is very similar to the HO phase, with a time complexity which is consistent with HO. In the small-hole imaging reverse learning phase, the time complexity of this phase, denoted as $T_{3}^{\prime }=O\left(P\times D\right)$, is executed in each iteration, resulting in a total complexity of $O\left(T\times P\times D\right)$. Thus, the overall time complexity of the MHO algorithm can be summarized as $T_{MHO}=T_{1}^{\prime }+T_{2}+T_{3}^{\prime }$ with a final time complexity of $O\left(P\right)$. It is worth noting that the time complexity of MHO is comparable to HO, which indicates that the enhancement strategy proposed in this study does not affect the solution efficiency of the algorithm.

## 3. Experiment

In this section, a series of experiments are designed to validate the effectiveness of the HO improvement algorithm, and we have chosen 23 benchmark test functions to evaluate the MHO algorithm and to perform comparison experiments with nine other meta-heuristic algorithms. In addition, ablation experiments of the algorithm were conducted to explore the contribution and impact of different components in the MHO algorithm.

### 3.1. Experimental Setup and Evaluation Criteria

To ensure the fairness and validity of the experiments, this paper proposes that the HO-based improved algorithm MHO, as well as other nature-inspired algorithms, are programmed and implemented in an experimental environment on Windows 10, all on a computer configured with 12th Gen Intel (R) Core (TM) i5-12600KF 3.70 GHz processor, 16 Gb RAM, and using the programming language MATLAB 2019b. The performance of the algorithms is evaluated using the following evaluation criteria:

Mean: the average value computed by the algorithm after executing it several times for the benchmark function tested. The mean value indicates the general effectiveness of the algorithm in finding the optimal solution, i.e., the desired performance of the algorithm. A lower mean value indicates that the algorithm is able to find a better solution on average over multiple runs. The formula is calculated as in Equation (23):

(23) $$Mean=\frac{1}{S}\sum_{i=1}^{S}F_{i}$$

where S is the number of executions and $F_{i}$ denotes the result of the ith execution.

Standard deviation: the standard deviation calculated by the algorithm after executing the test functions many times. The smaller the standard deviation, the more stable the performance of the algorithm, which usually means that the algorithm has better robustness. The formula is shown in Equation (24):

(24) $$Std=\sqrt{\frac{1}{S}\sum_{i=1}^{S}{\left(F_{i}-\frac{1}{S}\sum_{i=1}^{S}F_{i}\right)}^{2}}$$

Rank: ranks the results of the Friedman test for all algorithms; the lower the mean and Std, the higher the rank. Algorithms with the same result are given comparative ranks to each other. “Rank-Count” represents the cumulative sum of the ranks, “Ave-Rank”’ represents the average of the ranks, and “Overall-Rank” is the final ranking of the algorithms in all comparisons.

### 3.2. Test Function

In order to test the improved performance of the MHO algorithm, 23 benchmark functions with different characteristics are used for testing and the specific function information is shown in Table 1, which contains the dimensionality (Dim), the domain, and the known theoretical optimum of the function. These test functions are grouped into three categories: single-peak test functions for $f_{1}∼f_{7}$, multimodal functions for $f_{8}∼f_{13}$, and fixed-dimension functions for $f_{14}∼f_{23}$. The single-peak benchmark function is characterized by the existence of only one global optimum solution and is monotonic and deterministic, so it is suitable for evaluating the speed of convergence and the development capability of optimization algorithms. The multimodal function has multiple local optimal solutions but only one global optimal solution, which makes it commonly used to test the global search capability of optimization algorithms and their ability to avoid falling into local optima. Fixed-dimension multimodal functions, on the other hand, are usually defined in a specific dimension, meaning that their complexity and difficulty are fixed and do not change as the dimension changes. This ensures consistency and comparability of tests.

### 3.3. Sensitivity Analysis

MHO is a population-based optimizer that performs the optimization process through iterative computation. Therefore, it can be expected that the experimental results are usually influenced by the number of fitness evaluations ($FE_{s}=P*t$), where P is the population size and t is the number of iterations. Most of the studies in the literature fix $FE_{s}$ at 15,000 iterations, i.e., when P=30 and t=500. However, different P and t settings can have an impact on the algorithm’s performance. Therefore, we chose three different p/t combinations (20/75000, 30/500, and 60/250) to analyze their effects on the MHO algorithm. Seventeen test functions were randomly selected for sensitivity analysis, and the experimental results are shown in Table 2.

As can be seen in Table 2, for $f_{7},\;f_{12},\;f_{20}$ the best results for these three functions are achieved for four different p/t settings. For $f_{16},\;f_{19},\;f_{20},\;f_{21},\;f_{23}$ of these six functions, the p/t of 20/750 has the smallest value of standard deviation. p/t of 30/500 for the functions $f_{14}$ and $f_{17}$ exhibits smaller values of Std. Rank-Count is the sum of the rank values of all functions for the same set of p/t, where the Rank-Count value of 32.5 for p/t of 30/500 is the smallest. After the Friedman test, it can be seen that the first place on the final ranking (Overall-Rank) is p/t of 30/500, so it can be concluded that this experimental result is the best and is set as a fixed parameter for the experiment in this paper.

### 3.4. Experimental Results

Comparative experiments were conducted on the above twenty-three test functions for HO as well as variants of HO (HO1, HO2, and HO3) and comparing them with the Harris hawk algorithm (HHO) [49], honey badger algorithm (HBA) [50], dung beetle optimization algorithm (DBO) [51], particle swarm optimization algorithm (PSO), and whale optimization algorithm (WOA) [52], where HO1 is the HO with the introduction of the sine chaotic map after HO, using Equation (4) to replace the population initialization method, HO2 incorporates the small-hole imaging reverse learning strategy, using Equation (17) to add a reverse learning process, and HO3 is to improve the growth mechanism of HO (Equation (13)). The evaluation process is set with uniform parameters to ensure fairness, and each algorithm will perform 50 cycles each time. The experimental results are shown in Table 3.

Observing the data in Table 3 yields the performance of our MHO algorithm and its comparative algorithms on several benchmark functions. The MHO algorithm outperforms the other algorithms in terms of mean and standard deviation on the functions $f_{1}∼f_{4}$ and is slightly inferior to the HHO algorithm on the functions $f_{5}∼f_{6}$, but the MHO algorithm is second only to the HHO algorithm in terms of mean and standard deviation on the function $f_{6}$. The MHO algorithm is superior to all other algorithms except the variant HO2 on the function $f_{7}$. For the multimodal benchmark function $f_{8}∼f_{13}$, the MHO algorithm performs optimally on the function $f_{9},\;f_{10},\;f_{11}$. It is inferior to the HHO on both the $f_{12}$ and $f_{13}$ functions, but the mean value of $f_{13}$ is second only to the HHO and is superior to the other algorithms. For the fixed-dimensional test functions $f_{14}∼f_{23}$, it outperforms the other algorithms in terms of mean and standard deviation for the six test functions $f_{14}$, $f_{15}$ and $f_{20}∼f_{23}$, and while the standard deviation is slightly worse than the other algorithms for the four functions, the mean values are optimal. For the fixed-dimensional test functions $f_{14}∼f_{23}$, MHO outperforms the other algorithms in terms of mean and standard deviation for the six test functions, while the standard deviation is slightly worse than the other algorithms for the four functions $f_{16}∼f_{19}$, but the mean values are optimal. 

Summarizing the above results, it can be seen that the MHO algorithm shows a clear advantage in the benchmark function. Whether on single-peak, multi-peak, or hybrid functions, it shows excellent optimization performance and stability. These results fully demonstrate the effectiveness and superiority of the MHO algorithm in solving complex optimization problems.

### 3.5. Friedman Test

The Friedman test [53] provides an effective tool for performance comparison of optimization algorithms, statistical significance analysis, robustness assessment, and multi-objective optimization, which allows us to make scientific and reasonable algorithm selections and applications. Through the Friedman test, we can fairly compare the performance of different algorithms and reduce the bias caused by the selection of specific problems, so as to make objective evaluations and scientific decisions.

Therefore, in order to further compare the overall performance of these 10 algorithms, the algorithms are ranked using the Friedman test, and Table 4 shows the performance rankings of the 10 algorithms, including MHO, on 17 randomly selected functions out of the 23 benchmark functions mentioned above. From the table, it can be concluded that MHO has a sum of 50.5 ranking numbers, an average ranking of 2.1597, and a final ranking of 1, which indicates that MHO has the best overall performance. The results of the Friedman test once again prove that MHO performs better than the other algorithms.

### 3.6. Convergence Analysis

The convergence curve usually indicates the process of the algorithm gradually approaching the optimal solution during the iteration process, which can simply and intuitively show the advantages and disadvantages of one or more algorithms, so the convergence analysis is a key step in verifying whether the MHO algorithm can stably find the optimal solution or near-optimal solution to the optimization problem.

**Figure 5:**
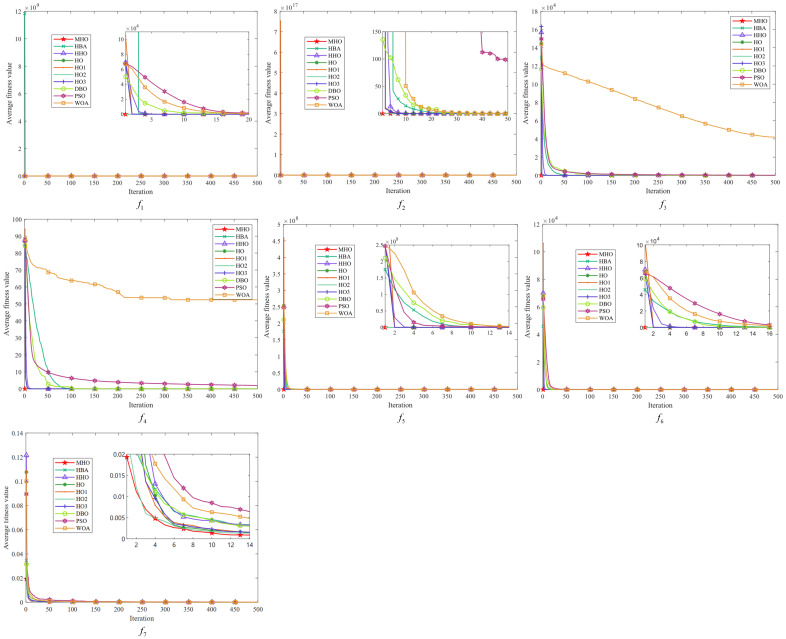
Convergence plots of single-peak function.

**Figure 6:**
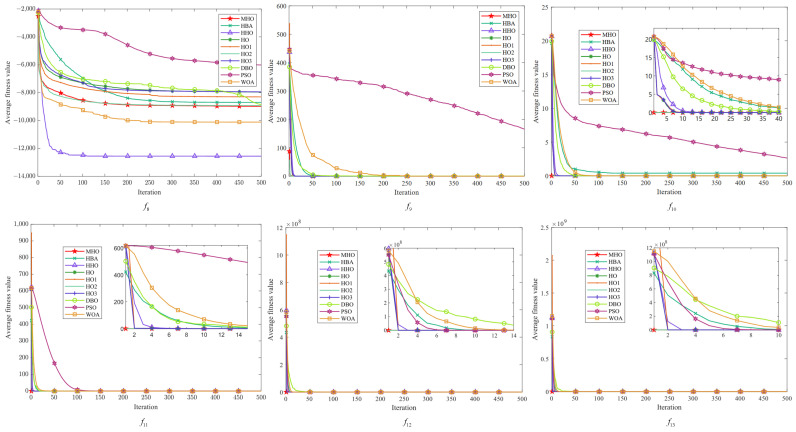
Convergence plots of multi-peak function.

**Figure 7:**
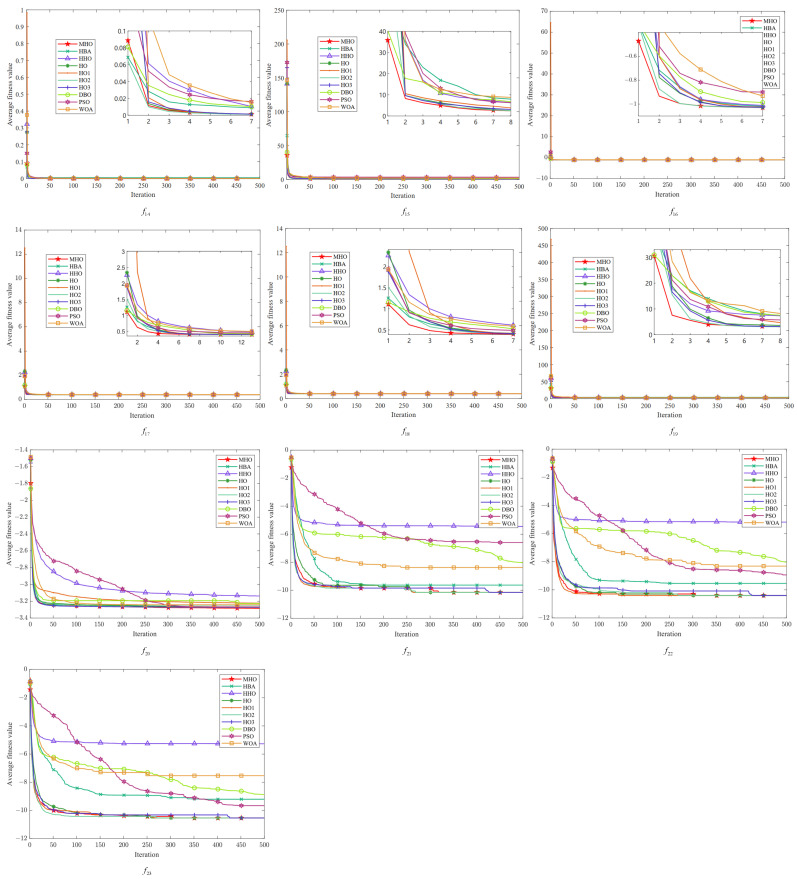
Convergence plots of fixed-dimensional multimodal function.

In this study, the average fitness value of the objective function is used as the criterion for evaluating the convergence of the algorithms, and each algorithm is iterated up to 500 times. We visualize the experimental results of ten algorithms, including MHO, on 23 benchmark functions, and the obtained convergence curves are subjected to convergence analysis. As shown in Figure 5, Figure 6 and Figure 7, the convergence plots are shown for the single-peak function, the multimodal function, and the fixed-dimension function, respectively.

All the convergence curves of the single-peak function are shown in Figure 5. The initial solution of MHO is always the lowest among the convergence curves on these seven functions, indicating that it is able to find a good quality solution at the initial stage. Among them, except for $f_{7}$, the variant HO2 has similar curves to MHO, and the convergence speed as well as the accuracy is optimal, which reflects the effectiveness of the reverse learning strategy for small-aperture imaging. All the curves converge to the same level; except for the PSO of $f_{2}$ and the WOA of $f_{3}$, all curves tend to converge. On the $f_{7}$ function, the convergence speed of MHO is not similar to other algorithms, but the value of its optimal solution is the smallest, so the overall performance is better than other algorithms.

All convergence curves for the multi-peak function are shown in Figure 6. Again, the curves of MHO and HO2 are similar on the six functions on the graphs. On the $f_{8}$ function, the fitness values of MHO and other functions are significantly lower than those of HHO, but on all functions other than that, the dominance of MHO is similar to that on the single-peak function. On $f_{9}$, it can be seen that MHO and the green line of HO2 converge preferentially, followed by four similar lines for HO, HO1, HO3, and HHO converging one after another; PSO shows the worst convergence rate and fitness values on $f_{8}∼f_{11}$.

Figure 7 shows the multimodal function with fixed dimensions. There are few overall differences between all the algorithms in function $f_{14}∼f_{19}$, but there are noticeable differences between the curves in the detailed presentation. The same characteristics of MHO are exhibited in all these functions—a rapid decline in the initial period, showing a fast rate of convergence—and the other algorithms also show faster convergence on specific functions, but with lower fitness values for MHO. Among the functions $f_{20}∼f_{23}$, HHO has the worst overall performance, and MHO shows good optimization performance with fast convergence speed and optimal solutions on all functions. The other algorithms also perform well on specific functions but, overall, the MHO algorithm shows competitiveness in these tests.

### 3.7. Stability Analysis

**Figure 8:**
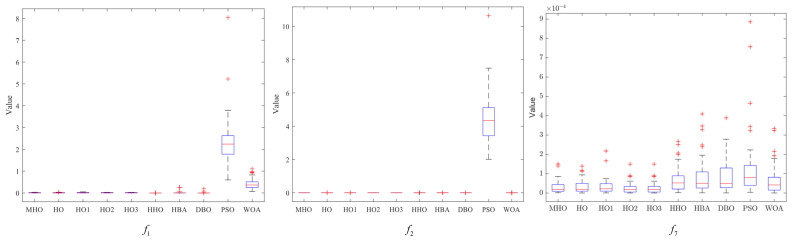
Boxplots of single-peak function.

**Figure 9:**
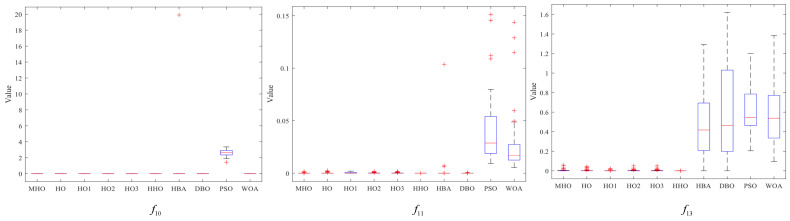
Boxplots of multi-peak function.

**Figure 10:**
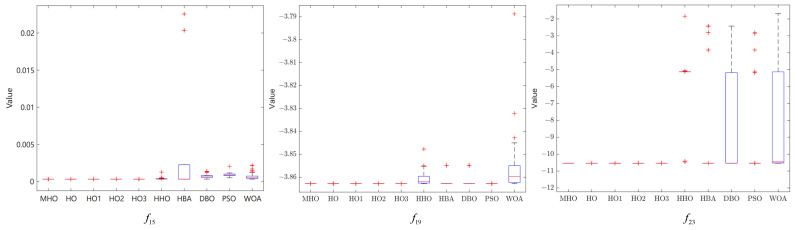
Boxplots of multimodal functions with fixed dimensions.

In this section, box-and-line plots are used to analyze the stability of all the algorithms, which are run independently 50 times, again using the experimental results for the 23 benchmark functions. Figure 8, Figure 9 and Figure 10 show the box-and-line plots for the single-peak function, the multi-peak function, and the fixed-dimension multimodal function, respectively. As an example, the boxplots of the functions in Figure 7 are presented as an evaluation method of the boxplots.

In the box-and-line plot, the red horizontal line represents the median, with lower values indicating better performance of the algorithm on the test function. It is the primary metric for evaluating the performance of the algorithms. MHO has a low median, with all the algorithms except HHO showing similar performance. The blue boxes for DBO and WOA show the interquartile range (IQR), where a smaller IQR indicates a more stable algorithmic performance. Thus, MHO, HO, HO1, HO2, HO3, HHO, HBA, and WOA all show better stability. The red crosses represent outliers, and the smaller the number, the better the stability. Here, only HHO, HBA, and PSO have outliers, implying that the other algorithms are more stable. The gray dotted line represents the whisker; the longer the whisker, the more discrete the data are. The longer whisker of DBO shows that it performs poorly. The stability of the algorithm can be analyzed by combining the above evaluation parameters. As a side note, we have chosen some representative examples to keep the data concise.

Among the single-peak functions shown in Figure 8, MHO has the lowest median, the smallest outliers, the smallest interquartile spacing, and shows better stability, while PSO is the least stable; the other four single-peak functions are consistent in their general trend with the representative cases shown. 

Among the multi-peak functions presented in Figure 9, MHO has the lowest median and performs better in terms of stability, with only slightly more outliers on $f_{11},\;f_{13}$ than HHO; among the functions not presented, the overall trend is consistent with the representative cases presented, with MHO being slightly weaker in terms of stability than HHO as well as PSO performing the worst.

Among the multimodal functions shown in Figure 10, MHO has the lowest median and outliers and the best stability, while HHO performs the worst. The general trend in the performance of the algorithms in the non-shown functions is consistent with the shown functions. The combined boxplot analysis of the above algorithms leads to the conclusion that MHO has the best stability.

## 4. Application to Engineering Design Problems

Three typical engineering constraint problems—reducer design [54], gear train design [55], and step taper pulley design [56]—are chosen for examination in this section in order to further confirm the efficacy of MHO in resolving global optimization issues. Because of their intricate restrictions and multi-objective optimization features, these issues are not only significant in engineering practice but also make excellent examples for evaluating the effectiveness of optimization methods. The trials are set up as 50 rounds of cycles with a maximum number of iterations per round of 50, and we will compare MHO’s performance with that of other algorithms to confirm its effectiveness.

### 4.1. Speed Reducer Design Problem

**Figure 11:**
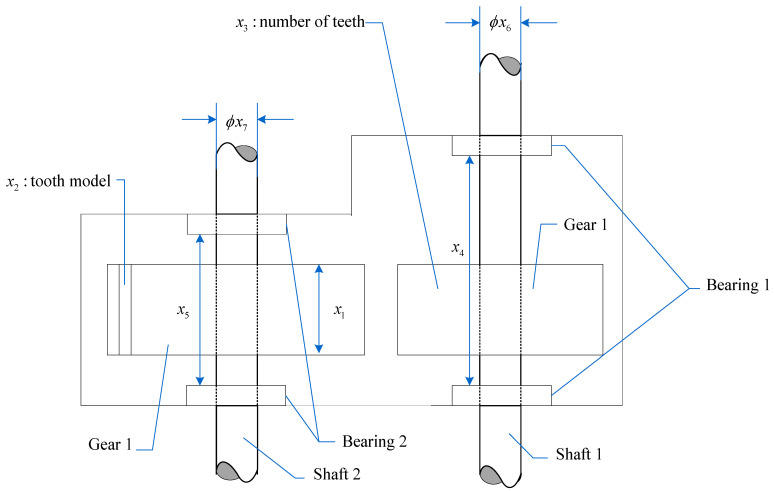
Speed reducer design problem diagram.

Reducers are key components in mechanical drive systems. As shown in Figure 11, the design of a speed reducer is challenging. This is because seven design variables are involved: face width ($x_{1}$), module of teeth ($x_{2}$), number of teeth on the pinion ($x_{3}$), length of the first shaft between the bearings ($x_{4}$), length of the second shaft between the bearings ($x_{5}$), diameter of the first shaft ($x_{6}$), and diameter of the second shaft ($x_{7}$). The objective is to minimize the total weight of the gearbox while satisfying 11 constraints. The constraints include bending stresses in the gear teeth, surface stresses, lateral deflections of shaft 1 and shaft 2 due to transmitted forces, and stresses in shaft 1 and shaft 2. The mathematical model is shown in Equation (25):

(25) $$\begin{array}{ll} Consider\; & \bar{x}=\left[x_{1},x_{2},x_{3},x_{4},x_{5},x_{6},x_{7}\right]=\left[b,m,p,l_{1},l_{2},d_{1},d_{2}\right]\\ Minimize\; & f(\bar{x})=0.7854x_{1}x_{2}^{2}\left(3.3333x_{3}^{2}+14.9334x_{3}-43.0934\right)-1.508x_{1}\left(x_{6}^{2}+x_{7}^{2}\right)\\ & \;\;\;+7.4777\left(x_{6}^{3}+x_{7}^{3}\right)+0.7854\left(x_{4}x_{6}^{2}+x_{5}x_{7}^{2}\right)\\ Subject\;to & g_{1}(\bar{x})=\frac{27}{x_{1}x_{2}^{2}x_{3}}-1⩽0,\\ & g_{2}(\bar{x})=\frac{397}{x_{1}x_{2}^{2}x_{3}^{2}}-1⩽0,\\ & g_{3}(\bar{x})=\frac{1.93x_{4}^{3}}{x_{2}x_{6}^{4}x_{3}}-1⩽0,\\ & g_{4}(\bar{x})=\frac{1.93x_{5}^{3}}{x_{2}x_{7}^{4}x_{3}}-1⩽0,\\ & g_{5}(\bar{x})=\frac{{\left[{\left(745\left(x_{4}/x_{2}x_{3}\right)\right)}^{2}+16.9\times 10^{6}\right]}^{1/2}}{110x_{6}^{2}}-1⩽0,\\ & g_{6}\left(\bar{x}\right)=\frac{{\left[{\left(745\left(x_{5}/x_{2}x_{3}\right)\right)}^{2}+157.9\times 10^{6}\right]}^{1/2}}{85x_{7}^{3}}-1⩽0,\\ & g_{7}(\bar{x})=\frac{x_{2}x_{3}}{40}-1⩽0,\\ & g_{8}(\bar{x})=\frac{5x_{2}}{x_{1}}-1⩽0,\\ & g_{9}(\bar{x})=\frac{x_{1}}{12x_{2}}-1⩽0,\\ & g_{10}(\bar{z})=\frac{1.5z_{6}+1.9}{z_{4}}-1⩽0,\\ & g_{11}(z)=\frac{1.5z_{7}+1.9}{z_{5}}-1⩽0,\\ where & \\ & 2.6⩽x_{1}⩽3.6,\;0.7⩽x_{2}⩽0.8,\;17⩽x_{3}⩽28,\;7.3⩽x_{4}⩽8.3,\\ & 7.3⩽z_{5}⩽8.3,\;\;\;2.9⩽x_{6}⩽3.9,\;5.0⩽x_{7}⩽5.5. \end{array}$$

MHO is compared to nine other algorithms in order to address the speed reducer design problem. Table 5 displays the results of the experiment, and it is evident that MHO is the least costly algorithm.

### 4.2. Gear Train Design Problem

**Figure 12:**
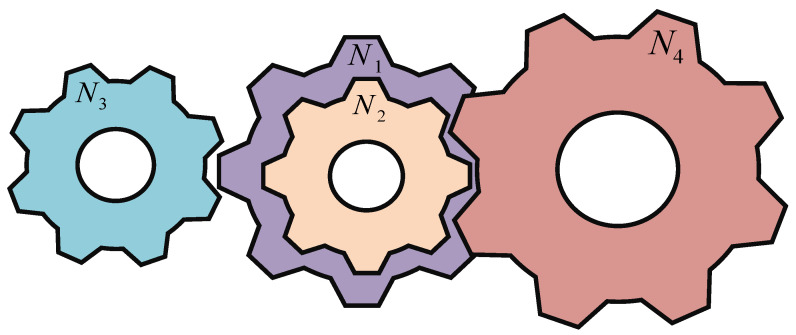
Gear system design problem diagram.

The gear train design problem aims to minimize the cost of the gear ratios shown in Figure 12. This problem has four integer decision variables, where $N_{1}$, $N_{2}$, $N_{3}$, and $N_{4}$ represent the number of teeth of four different gears. The mathematical model is shown in Equation (26):

(26) $$\begin{array}{l} Consider\;\vec{x}=\left[x_{1},x_{2},x_{3},x_{4}\right]=\left[N_{1},N_{2},N_{3},N_{4}\right]\\ Minimize\;f\left(\vec{x}\right)={\left(\frac{1}{6.931}-\frac{x_{2}x_{3}}{x_{1}x_{4}}\right)}^{2},\\ Variable\;range\;12\leq x_{i}\leq 60,\;i=1,2,3,4. \end{array}$$

The MHO algorithm is employed to optimize the design of gear systems, and its results are compared with those of nine other algorithms. The experimental results are shown in Table 6. The optimal value obtained by MHO is lower than that of the other nine algorithms, indicating that MHO achieved a better value and superior performance in this problem.

### 4.3. Step-Cone Pulley Problem

**Figure 13:**
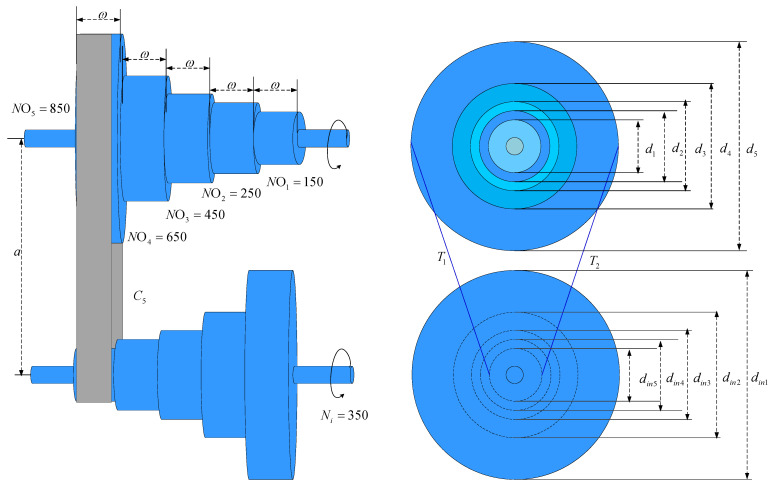
Step-cone pulley problem diagram.

A stepped conical pulley is a pulley consisting of two or more conical pulleys connected as shown in Figure 13 with five design variables: $d_{i}$, the diameter of the pulley at step $i\in \left[1,4\right]$, and ω, the width of the belt and the pulleys at each step. The goal of the system is to minimize the weight of the step conical pulley, and the problem contains 11 nonlinear constraints to ensure that the transmission power is at least 0.75 hp. Equation (27) is the mathematical model of the stepped conical pulley problem:

(27) $$\begin{array}{ll} Consider\; & \vec{x}=\left[x_{1},x_{2},x_{3},x_{4},x_{5}\right]=\left[d_{1},d_{2},d_{3},d_{4},\omega \right]\\ Minimize\; & f(\bar{x})=\rho x_{5}\left[x_{1}^{2}\left\{11+{\left(\frac{N_{1}}{N}\right)}^{2}\right\}+x_{2}^{2}\left\{1+{\left(\frac{N_{2}}{N}\right)}^{2}\right\}+x_{3}^{2}\left\{1+{\left(\frac{N_{3}}{N}\right)}^{2}\right\}\right.\left.+x_{4}^{2}\left\{1+{\left(\frac{N_{4}}{N}\right)}^{2}\right\}\right]\\ Subject\;to\; & h_{1}(\bar{x})=C_{1}-C_{2}=0,\\ & h_{2}(\bar{x})=C_{1}-C_{3}=0,\\ & h_{3}(\bar{x})=C_{1}-C_{4}=0,\\ & g_{i=1,2,3.4}(\bar{x})=-R_{i}⩽2,\\ & g_{i=1,2,3.4}(\bar{x})=(0.75\times 745.6998)-P_{i}⩽0\\ where, & \\ & C_{i}=\frac{\pi x_{i}}{2}\left(1+\frac{N_{i}}{N}\right)+\frac{{\left(\frac{N_{i}}{N}-1\right)}^{2}}{4a}+2a,\;i=(1,2,3,4)\\ & R_{i}=\exp\left(\mu \left\{\pi -2\sin^{-1}\left\{\left(\frac{N_{i}}{N}-1\right)\frac{x_{i}}{2a}\right\}\right\}\right),\;i=(1,2,3,4),\\ & P_{i}=\mathrm{st}x_{5}\left(1-R_{i}\right)\frac{\pi x_{i}N_{i}}{60},\;i=(1,2,3,4),\\ & t=8\;\mathrm{mm},\;s=1.75\;\mathrm{MPa},\;\mu =0.35,\;\rho =7200\;\mathrm{kg}/m^{3},\;a=3\;\mathrm{mm}. \end{array}$$

The stepped tapered pulley problem is solved using MHO, and it is compared to nine alternative techniques. A maximum of 50 iterations and 50 training rounds were used in each experiment. MHO is best, according to the experiment results, which are displayed in Table 7. 

## 5. Conclusions and Outlook

In this paper, we propose a modified hippopotamus optimization algorithm that aims to further improve the algorithm’s performance and address the issue of the algorithm’s easy descent into local optima.

The introduction of the sine chaotic map to initialize the population improves the diversity and randomness of the hippopotamus population, which enables the hippopotamus optimization algorithm to achieve a better balance between global and local searching, thus improving the initial solution quality as well as the convergence speed.

Premature convergence can be avoided by optimizing the hippo’s position update technique with a new convergence factor. In addition, a small-hole imaging reverse learning strategy is incorporated to improve the performance of the algorithm by mapping the current optimal solution of the algorithm dimension by dimension, avoiding interference between the dimensions, and at the same time expanding the search range of the algorithm. 

Also, the proposed algorithm was experimented on with 23 test functions, and the performance of MHO was compared with HO and its variants as well as other metaheuristics, and the mean and standard deviation of the algorithm’s optimized search were calculated. The experimental results show that MHO is optimal in terms of mean and standard deviation for thirteen test functions, while failing to optimize in terms of mean and standard deviation for only five test functions. After analyzing the experimental results by using sensitivity analysis and the Friedman test for stability and convergence, respectively, it is concluded that MHO has a higher ranking and stability and can jump out of local optima faster. In order to further verify the ability of MHO in solving global optimization problems, it is applied to three engineering design problems and compared with other algorithms, and the results show that MHO obtains very impressive outcomes. The above experiments fully demonstrate that compared with other existing algorithms, MHO possesses a stronger global search capability and is able to explore the solution space more efficiently, thus searching for potential optimal solutions more comprehensively. In addition, MHO significantly improves its adaptability to complex optimization problems by dynamically adjusting the search direction and step size, thus achieving faster convergence. In terms of local searching, MHO is able to locate the optimal solution more accurately, especially for high-dimensional complex optimization problems, and its unique mechanism enables it to avoid falling into the local optimal trap. MHO also demonstrates higher robustness and outperforms the other nine compared algorithms in both parameter optimization and real engineering problems.

Nevertheless, MHO still has a tendency to converge to locally optimal solutions for certain functions when working with global optimization issues. On the complicated reducer design challenges, MHO’s solution performance is also not very steady. Therefore, we will continue to improve the exploration and production capability of MHO in our future research. Meanwhile, we will apply MHO to a wider range of problems, such as multi-objective optimization and current popular neural networks.

## Figures and Tables

**Table 1:** Benchmark functions.

Function	Dimension	Domain	Theoretical Optimum
$f_{1}(x)=\sum_{i=1}^{n}x_{i}^{2}$	30	[−100,100]	0
$f_{2}(x)=\sum_{i=1}^{n}\left|x_{i}\right|+\prod_{i=1}^{n}\left|x_{i}\right|$	30	[−10,10]	0
$f_{3}(x)=\sum_{i=0}^{n-1}{\left\{\sum_{j=0}^{j<i}x_{i}\right\}}^{2}$	30	[−100,100]	0
$f_{4}(x)=\max_{i}\left\{\left|x_{i}\right|,\;1\leq i\leq n\right\}$	30	[−100,100]	0
$f_{5}(x)=\sum_{i=1}^{n-1}\left[100{\left(x_{i+1}-x_{i}^{2}\right)}^{2}+{\left(x_{i}-1\right)}^{2}\right]$	30	[−30,30]	0
$f_{6}(x)={\sum_{i=1}^{n}\left(\left[x_{i}+0.5\right]\right)}^{2}$	30	[−100,100]	0
$f_{7}(x)=\sum_{i=1}^{n}ix_{i}^{4}+random\left[0,1\right)$	30	[−1.28,1.28]	0
$f_{8}(x)=\sum_{i=1}^{n}-xi\sin\left(\sqrt{\left|x_{i}\right|}\right)$	30	[−500,500]	−12,569.4
$f_{9}(x)=\sum_{i=1}^{n}\left[x_{i}^{2}-10\cos\left(2\pi x_{i}\right)+10\right]$	30	[−5.12,5.12]	0
$\begin{array}{l} f_{10}(x)=-20\exp\left(-0.2\sqrt{\left(1/n\right)\times \sum_{i=1}^{n}x_{i}^{2}}\right)\\ -\exp\left(\left(1/n\right)\times \sum_{i=1}^{n}\cos\left(2\pi x_{i}\right)\right)+20+e \end{array}$	30	[−32,32]	0
$f_{11}=\left(1/4000\right)\times \sum_{i=1}^{n}x_{i}^{2}-\prod_{i=1}^{n}\cos\left(x_{i}/\sqrt{x+1}\right)+1$	30	[−600,600]	0
$\begin{array}{ll} f_{12}(x)= & \left(\pi /n\right)\times \left\{10\sin\left(\pi y_{1}\right)+\right.{\left(y_{n}-1\right)}^{2}\\ & +\sum_{i=1}^{n-1}{\left(y_{i}-1\right)}^{2}\left[1+10\sin^{2}\left(\pi y_{i+1}\right)\right]\} \end{array}\;\begin{array}{l} y_{i}=1+\left(x_{i}+1\right)/4 \end{array}\;\begin{array}{l} u\left(x_{i},a,k,m\right)=\left\{\begin{array}{cc} k{\left(x_{i}-a\right)}^{m} & x_{i}>a\\ 0 & -a<x_{i}<1\\ k{\left(-x_{i}-a\right)}^{m} & x_{i}<-a \end{array}\right. \end{array}$	30	[−50,50]	0
$\begin{array}{ll} f_{13}(x)= & 0.1\{\sum_{i=1}^{n}{\left(x_{i}-1\right)}^{2}\left[1+\sin^{2}\left(3\pi x_{1}+1\right)\right]\\ & \sin^{2}\left(3\pi x_{1}\right)+{\left(x_{n}-1\right)}^{2}\left[1+\sin^{2}\left(2\pi x_{ni}\right)\right]\}\\ & +\sum_{i=1}^{n}u\left(x_{i},5,100,4\right) \end{array}$	30	[−50,50]	0
$f_{14}(x)={\left(1/500+\sum_{j=1}^{25}\left(1/\left(j+\sum_{i=1}^{2}{\left(x_{i}-a_{ij}\right)}^{6}\right)\right)\right)}^{-1}$	2	[−65,65]	1
$f_{15}(x)={\sum_{i=1}^{11}\left[a_{i}-x_{1}\left(b_{i}^{2}+b_{i}x_{2}\right)/\left(b_{i}^{2}+b_{i}x_{3}+x_{4}\right)\right]}^{2}$	4	[−5,5]	0.00003075
$f_{16}(x)=4x_{1}^{2}-2.1x_{1}^{4}+x_{1}^{6}/3+x_{1}x_{2}-4x_{2}^{2}+4x_{2}^{4}$	2	[−5,5]	−1.0316285
$\begin{array}{ll} f_{17}(x)= & {\left(x_{2}-\left(5.1/4\pi^{2}\right)x_{1}^{2}+\left(5/\pi \right)x_{1}-6\right)}^{2}\\ & +10\left(1-\left(1/8\pi \right)\right)\cos x_{1}+10 \end{array}$	2	[−5,5]	0.398
$\begin{array}{ll} f_{18}(x)= & \left[1+{\left(x_{1}+x_{2}+1\right)}^{2}\times \left(19-14x_{1}+3x_{1}^{2}-14x_{2}+6x_{1}x_{2}+3x_{2}^{2}\right)\right]\\ & \cdot \left[30+{\left(2x_{1}-3x_{2}\right)}^{2}\times \left(18-32x_{1}+12x_{1}^{2}+48x_{2}-36x_{1}x_{2}+27x_{2}^{2}\right)\right] \end{array}$	2	[−2,2]	3
$f_{19}(x)=-\sum_{i=1}^{4}c_{i}\exp\left(-\sum_{j=1}^{3}a_{ij}{\left(x_{j}-p_{ij}\right)}^{2}\right)$	3	[0,1]	−3.86
$f_{20}(x)=-\sum_{i=1}^{4}c_{i}\exp\left(-\sum_{j=1}^{6}a_{ij}{\left(x_{j}-p_{ij}\right)}^{2}\right)$	6	[0,1]	−3.32
$f_{21}(x)=-{\sum_{i=1}^{5}\left[\left(x-a_{i}\right){\left(x-a_{i}\right)}^{T}+c_{i}\right]}^{-1}$	4	[0,10]	−10
$f_{22}(x)=-{\sum_{i=1}^{7}\left[\left(x-a_{i}\right){\left(x-a_{i}\right)}^{T}+c_{i}\right]}^{-1}$	4	[0,10]	−10
$f_{23}(x)=-{\sum_{i=1}^{10}\left[\left(x-a_{i}\right){\left(x-a_{i}\right)}^{T}+c_{i}\right]}^{-1}$	4	[0,10]	−10

**Table 2:** Sensitivity analysis of p/t.

Function	Criterion	p/t	p/t	p/t
		20/750	30/500	60/250
$f_{1}$	Mean	**0.0000**	**0.0000**	**0.0000**
	Std	**0.0000**	**0.0000**	**0.0000**
	Rank	2	2	2
$f_{2}$	Mean	**0.0000**	**0.0000**	**0.0000**
	Std	**0.0000**	**0.0000**	**0.0000**
	Rank	2	2	2
$f_{3}$	Mean	**0.0000**	**0.0000**	**0.0000**
	Std	**0.0000**	**0.0000**	**0.0000**
	Rank	2	2	2
$f_{4}$	Mean	**0.0000**	**0.0000**	**0.0000**
	Std	**0.0000**	**0.0000**	**0.0000**
	Rank	2	2	2
$f_{7}$	Mean	3.2851 × 10^−5^	2.8911 × 10^−5^	**2.7563 × 10^−5^**
	Std	3.4492 × 10^−5^	3.1692 × 10^−5^	**2.6425 × 10^−5^**
	Rank	3	2	1
$f_{9}$	Mean	**0.0000**	**0.0000**	**0.0000**
	Std	**0.0000**	**0.0000**	**0.0000**
	Rank	2	2	2
$f_{10}$	Mean	9.5923 × 10^−16^	**8.8818 × 10^−16^**	**8.8818 × 10^−16^**
	Std	5.0243 × 10^−16^	**0.0000**	**0.0000**
	Rank	3	1.5	1.5
$f_{11}$	Mean	**0.0000**	**0.0000**	**0.0000**
	Std	**0.0000**	**0.0000**	**0.0000**
	Rank	2	2	2
$f_{12}$	Mean	3.6758 × 10^−4^	**1.5255 × 10^−4^**	2.9019 × 10^−4^
	Std	5.1790 × 10^−4^	**3.5965 × 10^−4^**	4.3157 × 10^−4^
	Rank	3	1	2
$f_{14}$	Mean	**9.9800 × 10^−1^**	**9.9800 × 10^−1^**	**9.9800 × 10^−1^**
	Std	2.7532 × 10^−13^	**1.5468 × 10^−13^**	5.5052 × 10^−13^
	Rank	2	2	2
$f_{16}$	Mean	**−1.0316**	**−1.0316**	**−1.0316**
	Std	**3.2394 × 10^−11^**	3.6381 × 10^−10^	1.5708 × 10^−9^
	Rank	2	2	2
$f_{17}$	Mean	**3.9789 × 10^−1^**	**3.9789 × 10^−1^**	**3.9789 × 10^−1^**
	Std	4.4950 × 10^−10^	**3.9781 × 10^−10^**	2.7622 × 10^−9^
	Rank	2	2	2
$f_{19}$	Mean	**−3.8628**	**−3.8628**	**−3.8628**
	Std	**1.1802 × 10^−8^**	5.0575 × 10^−8^	6.2302 × 10^−8^
	Rank	2	2	2
$f_{20}$	Mean	**−3.2904**	**−3.2849**	**−3.2794**
	Std	**5.4536 × 10^2^**	5.8998 × 10^2^	6.7029 × 10^2^
	Rank	1	2	3
$f_{21}$	Mean	**−1.0153 × 10^1^**	**−1.0153 × 10^1^**	**−1.0153 × 10^1^**
	Std	**8.8671 × 10^−7^**	1.7912 × 10^−6^	3.8080 × 10^−6^
	Rank	2	2	2
$f_{22}$	Mean	**−1.0403 × 10^−1^**	**−1.0403 × 10^−1^**	**−1.0403 × 10^−1^**
	Std	**6.7735 × 10^−7^**	1.5216 × 10^−6^	6.3626 × 10^−6^
	Rank	2	2	2
$f_{23}$	Mean	−1.053 × 10^1^	**−1.056 × 10^1^**	**−1.056 × 10^1^**
	Std	**7.0815 × 10^−7^**	1.5856 × 10^−6^	9.3393 × 10^−6^
	Rank	2	2	2
Rank-Count		36	32.5	33.5
Ave-Rank		2.11	1.91	1.97
Overall-Rank		3	1	2

**Table 3:** Experimental results of MHO and its comparison algorithms.

Function	Algorithm	Mean	Std	Function	Algorithm	Mean	Std
$f_{1}$	MHO	**0.0000**	**0.0000**	$f_{13}$	MHO	5.2459 × 109^−3^	1.2318 × 10^−2^
	HO	0.0000	0.0000		HO	4.0138 × 108^−3^	9.4581 × 10^−3^
	HO1	0.0000	0.0000		HO1	1.7004 × 104^−3^	4.5484 × 10^−3^
	HO2	0.0000	0.0000		HO2	4.3263 × 103^−3^	9.7711 × 10^−3^
	HO3	0.0000	0.0000		HO3	1.0953 × 10^−2^	1.4091 × 10^−2^
	HHO	3.3722 × 10^−93^	2.3825 × 10^−92^		HHO	**9.9801 × 10^−5^**	**1.3388 × 10^−4^**
	HBA	6.9026 × 10^−72^	3.3528 × 10^−71^		HBA	4.6997 × 10^−1^	3.3318 × 10^−1^
	DBO	5.1200 × 10^−113^	2.6559 × 10^−112^		DBO	5.9622 × 102^−1^	4.7776 × 10^−1^
	PSO	2.5274	1.0479		PSO	6.1253 × 103^−1^	2.3179 × 10^−1^
	WOA	1.0234 × 10^−72^	6.5245 × 10^−72^		WOA	5.7769 × 10^−1^	3.1337 × 10^−1^
$f_{2}$	MHO	**0.0000**	**0.0000**	$f_{14}$	MHO	**9.9800 × 10^−1^**	**1.5468 × 10^−13^**
	HO	4.0358 × 10^−182^	0.0000		HO	9.9800 × 10^−1^	3.0556 × 10^−13^
	HO1	2.8707 × 10^−182^	0.0000		HO1	9.9800 × 10^−1^	2.4026 × 10^−13^
	HO2	0.0000	0.0000		HO2	9.9800 × 10^−1^	1.2184 × 10^−12^
	HO3	1.1752 × 10^−180^	0.0000		HO3	9.9800 × 10^−1^	8.5786 × 10^−12^
	HHO	9.2603 × 10^−51^	4.8939 × 10^−50^		HHO	1.6880	1.7750
	HBA	2.9432 × 10^−72^	1.2928 × 10^−71^		HBA	1.3517	1.5159
	DBO	1.5566 × 10^−54^	1.1007 × 10^−53^		DBO	1.4741	8.7881 × 10^−1^
	PSO	4.5005	1.4881		PSO	3.3460	2.3845
	WOA	8.9092 × 10^−50^	5.0084 × 10^−49^		WOA	2.1392	2.3978
$f_{3}$	MHO	**0.0000**	**0.0000**	$f_{15}$	MHO	**3.0751 × 10^−4^**	**4.0690 × 10^−8^**
	HO	0.0000	0.0000		HO	3.0752 × 10^−4^	1.0325 × 10^−7^
	HO1	0.0000	0.0000		HO1	3.0755 × 10^−4^	3.2657 × 10^−7^
	HO2	0.0000	0.0000		HO2	3.0752 × 10^−4^	4.7547 × 10^−8^
	HO3	0.0000	0.0000		HO3	3.0753 × 10^−4^	1.1442 × 10^−7^
	HHO	3.1810 × 10^−72^	2.2493 × 10^−71^		HHO	3.7963 × 10^−4^	1.8487 × 10^−4^
	HBA	3.2286 × 10^−93^	2.2092 × 10^−92^		HBA	4.9321 × 10^−3^	8.4966 × 10^−3^
	DBO	6.6703 × 10^−40^	4.7166 × 10^−39^		DBO	7.2540 × 10^−4^	3.1412 × 10^−4^
	PSO	1.7653 × 10^2^	4.5978 × 10^1^		PSO	8.9744 × 10^−4^	2.2012 × 10^−4^
	WOA	4.1941 × 10^4^	1.5808 × 10^4^		WOA	6.9236 × 10^−4^	4.7036 × 10^−^^4^
$f_{4}$	^MHO^	0.0000	0.0000	$f_{16}$	^MHO^	−1.0316	3.6381 × 10^−10^
	HO	2.8900 × 10^−184^	0.0000		HO	−1.0316	2.6830 × 10^−10^
	HO1	6.8888 × 10^−181^	0.0000		HO1	−1.0316	1.5659 × 10^−10^
	HO2	0.0000	0.0000		HO2	−1.0316	6.7215 × 10^−11^
	HO3	5.9113 × 10^−179^	0.0000		HO3	−1.0316	6.3616 × 10^−11^
	HHO	8.0996 × 10^−49^	5.6060 × 10^−48^		HHO	−1.0316	4.6598 × 10^−9^
	HBA	1.9110 × 10^−56^	7.6981 × 10^−56^		HBA	−1.0316	**3.2812 × 10^−16^**
	DBO	1.2250 × 10^−49^	8.6389 × 10^−49^		DBO	−1.0316	3.3269 × 10^−16^
	PSO	1.9645	2.5026 × 10^−1^		PSO	−1.0316	4.2439 × 10^−16^
	WOA	5.2418 × 10^1^	2.5506 × 10^1^		WOA	−1.0316	7.1329 × 10^−10^
$f_{5}$	MHO	1.1509 × 10^−1^	1.7645 × 10^−1^	$f_{17}$	MHO	**3.9789 × 10^−1^**	3.9781 × 10^−10^
	HO	5.4281 × 10^−2^	8.3571 × 10^−2^		HO	3.9789 × 10^−1^	2.3634 × 10^−9^
	HO1	3.4397 × 10^−2^	6.5186 × 10^−2^		HO1	3.9789 × 10^−1^	2.4502 × 10^−9^
	HO2	6.2677 × 10^−2^	1.0042 × 10^−1^		HO2	3.9789 × 10^−1^	1.6152 × 10^−9^
	HO3	9.1590 × 10^−2^	1.1923 × 10^−1^		HO3	3.9789 × 10^−1^	1.8410 × 10^−9^
	HHO	**1.2253 × 10^−2^**	**1.8448 × 10^−2^**		HHO	3.9790 × 10^−1^	2.4452 × 10^−5^
	HBA	2.4033 × 10^1^	8.3381 × 10^−1^		HBA	3.9789 × 10^−1^	**3.3645 × 10^−16^**
	DBO	2.5734 × 10^1^	2.7141 × 10^−1^		DBO	3.9789 × 10^−1^	3.3645 × 10^−16^
	PSO	9.2719 × 10^2^	4.7898 × 10^2^		PSO	3.9789 × 10^−1^	3.3645 × 10^−16^
	WOA	2.7988 × 10^1^	4.9259 × 10^−1^		WOA	3.9790 × 10^−1^	1.2215 × 10^−5^
$f_{6}$	MHO	9.4915 × 10^−3^	9.7607 × 10^−3^	$f_{18}$	MHO	**3.0000**	8.0461 × 10^−9^
	HO	8.1739 × 10^−3^	1.1091 × 10^−2^		HO	3.0000	6.2997 × 10^−9^
	HO1	1.1798 × 10^−2^	1.3268 × 10^−2^		HO1	3.0000	1.4408 × 10^−9^
	HO2	8.6772 × 10^−3^	9.5263 × 10^−3^		HO2	3.0000	7.5526 × 10^−8^
	HO3	2.2698 × 10^−2^	1.0268 × 10^−2^		HO3	3.0000	2.8452 × 10^−9^
	HHO	**1.5709 × 10^−4^**	**2.4626 × 10^−4^**		HHO	3.0000	6.7092 × 10^−7^
	HBA	4.7001 × 10^−2^	9.6949 × 10^−2^		HBA	4.0800	5.3446 × 10+0
	DBO	6.2521 × 10^−3^	2.9742 × 10^−2^		DBO	3.0000	**2.4628 × 10^−15^**
	PSO	2.3410 × 10^+0^	1.1406 × 10^+0^		PSO	3.0000	5.3245 × 10^−15^
	WOA	4.1539 × 10^−1^	2.5095 × 10^−1^		WOA	3.0001	3.0702 × 10^−4^
$f_{7}$	MHO	2.8911 × 10^−5^	3.1692 × 10^−5^	$f_{19}$	MHO	**−3.8628**	5.0575 × 10^−8^
	HO	3.2820 × 10^−5^	3.3002 × 10^−5^		HO	-3.8628	4.5360 × 10^−8^
	HO1	3.5287 × 10^−5^	3.9834 × 10^−5^		HO1	−3.8628	1.2794 × 10^−8^
	HO2	**2.5891 × 10^−5^**	**2.8914 × 10^−5^**		HO2	−3.8628	3.8218 × 10^−8^
	HO3	3.6700 × 10^−5^	3.4275 × 10^−5^		HO3	−3.8628	2.8635 × 10^−8^
	HHO	6.9272 × 10^−5^	6.3855 × 10^−5^		HHO	−3.8607	2.8916 × 10^−3^
	HBA	8.5039 × 10^−5^	9.4110 × 10^−5^		HBA	−3.8615	2.9188 × 10^−3^
	DBO	8.2668 × 10^−5^	8.4546 × 10^−5^		DBO	−3.8615	2.9188 × 10^−3^
	PSO	1.2883 × 10^−4^	1.7180 × 10^−4^		PSO	−3.8628	**2.4066 × 10^−15^**
	WOA	6.6807 × 10^−5^	7.6012 × 10^−5^		WOA	−3.8564	1.1435 × 10^−2^
$f_{8}$	MHO	−8.9787 × 10^3^	2.1339 × 10^3^	$f_{20}$	MHO	**−3.2849**	**5.8998 × 10^−2^**
	HO	−7.9594 × 10^3^	1.5232 × 10^3^		HO	−3.2687	6.6724 × 10^−2^
	HO1	**−1.9759** × 10^4^	2.9040 × 10^3^		HO1	−3.2230	6.0775 × 10^−2^
	HO2	−8.9256 × 10^3^	2.0041 × 10^3^		HO2	−3.2752	6.3694 × 10^−2^
	HO3	−7.9414 × 10^3^	1.5311 × 10^3^		HO3	−3.2722	6.5502 × 10^−2^
	HHO	−1.2553 × 10^4^	**8.7389 × 10^1^**		HHO	−3.1380	9.6909 × 10^−2^
	HBA	−8.6957 × 10^3^	8.9116 × 10^2^		HBA	−3.2458	1.4477 × 10^−1^
	DBO	−8.8398 × 10^3^	1.7724 × 10^3^		DBO	−3.2273	1.2686 × 10^−1^
	PSO	−6.0159 × 10^3^	1.3308 × 10^3^		PSO	−3.2673	5.9858 × 10^−2^
	WOA	−1.0110 × 10^4^	1.7827 × 10^3^		WOA	−3.2425	9.8594 × 10^−2^
$f_{9}$	MHO	**0.0000**	**0.0000**	$f_{21}$	MHO	**−1.0153** × 10^1^	**1.7912 × 10^−6^**
	HO	0.0000	0.0000		HO	−1.0153 × 10^1^	3.3627 × 10^−6^
	HO1	0.0000	0.0000		HO1	−1.0153 × 10^1^	2.4596 × 10^−6^
	HO2	0.0000	0.0000		HO2	−1.0153 × 10^1^	2.7432 × 10^−6^
	HO3	0.0000	0.0000		HO3	−1.0153 × 10^1^	1.1416 × 10^−5^
	HHO	0.0000	0.0000		HHO	−5.4448	1.3442
	HBA	0.0000	0.0000		HBA	−9.6319	2.0943
	DBO	3.1725 × 10^−1^	1.9833		DBO	−8.0235	2.6724
	PSO	1.6470 × 10^+2^	3.3948 × 10^1^		PSO	−6.5915	3.2019
	WOA	2.2737 × 10^−15^	1.1252 × 10^−14^		WOA	−8.3749	2.6669
$f_{10}$	MHO	**8.8818 × 10^−16^**	**0.0000**	$f_{22}$	MHO	**−** **1.0403 × 10^1^**	**1.5216 × 10^−6^**
	HO	8.8818 × 10^−16^	0.0000		HO	−1.0403 × 10^1^	1.6082 × 10^−6^
	HO1	8.8818 × 10^−16^	0.0000		HO1	−1.0403 × 10^1^	2.2791 × 10^−6^
	HO2	8.8818 × 10^−16^	0.0000		HO2	−1.0403 × 10^1^	1.7314 × 10^−6^
	HO3	8.8818 × 10^−16^	0.0000		HO3	−1.0403 × 10^1^	2.3335 × 10^−5^
	HHO	8.8818 × 10^−16^	0.0000		HHO	−5.1867	7.2997 × 10^−1^
	HBA	3.9818 × 10^−1^	2.8156		HBA	−9.5440	2.3556
	DBO	8.8818 × 10^−16^	0.0000		DBO	−8.0311	2.7098
	PSO	2.6298	4.0400 × 10^−1^		PSO	−8.9425	2.5016
	WOA	4.9383 × 10^−15^	2.3816 × 10^−15^		WOA	−8.3157	2.8065
$f_{11}$	MHO	**0.0000**	**0.0000**	$f_{23}$	MHO	**−** **1.0536 × 10^1^**	**1.5856 × 10^6^**
	HO	0.0000	0.0000		HO	−1.0536 × 10^1^	1.9358 × 10^6^
	HO1	0.0000	0.0000		HO1	−1.0536 × 10^1^	1.6338 × 10^6^
	HO2	0.0000	0.0000		HO2	−1.0536 × 10^1^	2.1182 × 10^6^
	HO3	0.0000	0.0000		HO3	−1.0536 × 10^1^	2.4098 × 10^6^
	HHO	0.0000	0.0000		HHO	−5.2693	1.1627
	HBA	0.0000	0.0000		HBA	−9.2044	2.8850
	DBO	0.0000	0.0000		DBO	−8.8744	2.7522
	PSO	1.2258 × 10^−1^	4.9294 × 10^−2^		PSO	−9.6646	2.2171
	WOA	5.7736 × 10^−3^	2.8737 × 10^−2^		WOA	−7.5317	3.3854
$f_{12}$	MHO	1.5255 × 10^−4^	3.5965 × 10^−4^				
	HO	3.1657 × 10^−4^	6.1589 × 10^−4^				
	HO1	3.9595 × 10^−4^	6.2673 × 10^−4^				
	HO2	2.5974 × 10^−4^	4.5742 × 10^−4^				
	HO3	7.4742 × 10^−4^	6.2937 × 10^−4^				
	HHO	**6.7478 × 10^−6^**	**1.0577 × 10^−5^**				
	HBA	2.3525 × 10^−3^	1.4694 × 10^−2^				
	DBO	4.4967 × 10^−5^	1.3964 × 10^−4^				
	PSO	4.0556 × 10^−2^	3.2624 × 10^−2^				
	WOA	2.6887 × 10^−2^	2.8830 × 10^−2^				

**Table 4:** Performance ratings of MHO and its comparative algorithms.

Function	MHO	HO	HO1	HO2	HO3	HHO	HBA	DBO	PSO	WOA
$f_{1}$	3	3	3	3	3	7	9	6	10	8
$f_{2}$	1.5	4	3	1.5	5	8	6	7	10	9
$f_{3}$	3	3	3	3	3	7	6	8	9	10
$f_{4}$	1.5	3	4	1.5	5	8	6	7	9	10
$f_{6}$	3	4	5	2	6	1	8	7	10	9
$f_{9}$	4	4	4	4	4	4	4	9	10	8
$f_{10}$	4	4	4	4	4	4	10	4	9	8
$f_{11}$	4.5	4.5	4.5	4.5	4.5	4.5	4.5	4.5	10	9
$f_{12}$	3	5	6	4	7	1	8	2	10	9
$f_{14}$	3	3	3	3	3	8	7	6	10	9
$f_{15}$	1	3	5	2	4	6	10	7	8	9
$f_{16}$	5.5	5.5	5.5	5.5	5.5	5.5	5.5	5.5	5.5	5.5
$f_{19}$	3.5	3.5	3.5	3.5	3.5	9	7.5	7.5	3.5	10
$f_{20}$	1	5	6	2	4	10	8	9	3	7
$f_{21}$	3	3	3	3	3	9	6	8	10	7
$f_{22}$	3	3	3	3	3	10	6	9	7	8
$f_{23}$	3	3	3	3	3	10	7	8	6	9
Rank-Count	50.5	63.5	68.5	52.5	70.5	112	118.5	114.5	140	144.5
Ave-Rank	2.1957	2.7609	2.9783	2.2826	3.0652	4.8696	5.1522	4.9783	6.0870	6.2826
Overall-Rank	1	3	4	2	5	6	8	7	9	10

**Table 5:** Comparison of the results for the speed reducer design problem.

Algorithm	Optimal Value							Optimal Cost
	$x_{1}$	$x_{2}$	$x_{3}$	$x_{4}$	$x_{5}$	$x_{6}$	$x_{7}$	
MHO	3.5999	7.0000 × 10^−1^	1.7000 × 10^1^	8.3000	7.7978	3.3985	5.2935	**3.0614 × 10^3^**
HO	3.5145	7.0000 × 10^−1^	1.7000 × 10^1^	7.4885	7.9394	3.3538	5.4191	3.0942 × 10^3^
HO1	3.5473	7.0000 × 10^−1^	1.7000 × 10^1^	7.6056	7.9826	3.7487	5.2885	3.1373 × 10^3^
HO2	3.5747	7.0000 × 10^−1^	1.7000 × 10^1^	7.3000	7.8843	3.3548	5.4199	3.1155 × 10^3^
HO3	3.5147	7.0000 × 10^−1^	1.7000 × 10^1^	7.8114	8.1951	3.3515	5.4857	3.1476 × 10^3^
HHO	3.5034	7.0000 × 10^−1^	1.8508 × 10^1^	8.0993	7.8316	3.7984	5.3924	3.4771 × 10^3^
HBA	3.5000	7.0000 × 10^−1^	1.7000 × 10^1^	8.2194	7.9955	3.5891	5.2869	3.0754 × 10^3^
DBO	3.6000	7.0000 × 10^−1^	1.7000 × 10^1^	8.3000	7.7154	3.9000	5.2867	3.2093 × 10^3^
PSO	3.6000	7.0000 × 10^−1^	1.7000 × 10^1^	7.8063	8.3000	3.9000	5.2869	3.2164 × 10^3^
WOA	3.6000	7.1931 × 10^−1^	1.7000 × 10^1^	8.2999	7.8366	3.3518	5.2903	3.1389 × 10^3^

**Table 6:** Comparison of the results of gear train design problem.

Algorithm	Optimal Value				Optimal Cost
	$N_{1}$	$N_{2}$	$N_{3}$	$N_{4}$	
MHO	44	13	21	43	**1.5450 × 10^−10^**
HO	57	12	37	54	8.8876 × 10^−10^
HO1	59	15	21	37	3.0676 × 10^−10^
HO2	56	23	13	37	6.6021 × 10^−10^
HO3	55	12	37	56	1.5247 × 10^−8^
HHO	47	12	26	46	9.9216 × 10^−10^
HBA	34	15	17	52	2.3576 × 10^−9^
DBO	60	15	15	26	2.3576 × 10^−9^
PSO	57	37	12	54	8.8876 × 10^−10^
WOA	52	35	12	56	2.3576 × 10^−9^

**Table 7:** Comparison of the results of step-cone pulley problem.

Algorithm	Optimal Value					Optimal Cost
	$d_{1}$	$d_{2}$	$d_{3}$	$d_{4}$	ω	
MHO	3.9835 × 10^1^	5.4824 × 10^1^	7.3067 × 10^1^	8.7626 × 10^1^	8.8851 × 10^1^	**2.7377 × 10^92^**
HO	4.0922 × 10^3^	5.6309 × 10^1^	7.5110 × 10^1^	8.9975 × 10^1^	8.6176 × 10^1^	1.4460 × 10^93^
HO1	4.0863 × 10^1^	5.6226 × 10^1^	7.4988 × 10^1^	8.9873 × 10^1^	8.8590 × 10^1^	3.3731 × 10^92^
HO2	4.0683 × 10^1^	5.5973 × 10^1^	7.4724 × 10^1^	8.9455 × 10^1^	8.9458 × 10^1^	5.9786 × 10^93^
HO3	4.0427 × 10^1^	5.5560 × 10^1^	7.4205 × 10^1^	8.8981 × 10^1^	8.9309 × 10^1^	9.2168 × 10^93^
HHO	4.1957 × 10^1^	5.5823 × 10^1^	8.3645 × 10^1^	8.6757 × 10^1^	8.8616 × 10^1^	5.2464 × 10^97^
HBA	4.0818 × 10^1^	5.6155 × 10^1^	7.4881 × 10^1^	8.9761 × 10^1^	8.6001 × 10^1^	4.8097 × 10^92^
DBO	4.0928 × 10^1^	5.6330 × 10^1^	7.5113 × 10^1^	9.0000 × 10^1^	9.0000 × 10^1^	8.5877 × 10^92^
PSO	4.0147 × 10^1^	5.5184 × 10^1^	7.3648 × 10^1^	8.8431 × 10^1^	9.0000 × 10^1^	1.2714 × 10^94^
WOA	4.0969 × 10^1^	5.8129 × 10^1^	7.5737 × 10^1^	8.7173 × 10^1^	8.7229 × 10^1^	8.5726 × 10^96^

## Data Availability

The data generated from the analysis in this study can be found in this article. This study does not report the original code, which is available for academic purposes from the lead contact. Any additional information required to reanalyze the data reported in this paper is available from the lead contact upon request.
